# Assessing methodological quality of Russian clinical practice guidelines and introducing AGREE II instrument in Russia

**DOI:** 10.1371/journal.pone.0203328

**Published:** 2018-09-11

**Authors:** Dina Ayratova Lienhard, Lidiya Vacheslavovna Kisser, Liliya Eugenevna Ziganshina

**Affiliations:** 1 School of Life Sciences, Arizona State University, Tempe, Arizona, United States of America; 2 Department of the Organization of Providing Medicines and Medical Devices of the Ministry of Health of the Kaliningrad Region, Kaliningrad, Russian Federation; 3 Cochrane Russia, Research and Education Centre for Evidence-Based Medicine, Cochrane, Russia, and Department of Basic and Clinical Pharmacology, Kazan Federal University, Kazan, Tatarstan, Russian Federation; University of Utah Hospital, UNITED STATES

## Abstract

**Background:**

There are multiple organisations in Russia that publish clinical practice guidelines (CPGs). The demand for CPGs and appreciation of their role in healthcare provision has been steadily growing. However, quality and methodology of development of CPGs have not been systematically addressed.

**Aim:**

To analyse the quality of Russian-produced CPGs for surgical treatment of hepatic-pancreatic-biliary diseases.

**Methods:**

We searched Russian databases for CPGs, published between 2013 and 2017. We identified 6 relevant documents that met our inclusion criteria. We approached four experts in the field with written and verbal instructions on the use of the AGREE II questionnaire.

**Results:**

All six CPGs received the highest domain scores for the domain *Clarity of Presentation* (46%–80%). The lowest domain scores were for the domain *Editorial Independence* (6%-25%). Overall, the experts put the highest total sum scores to the CPG for treating chronic pancreatitis (70%), while the lowest total sum score was attributed to the CPG for treating acute cholangitis (22%).

**Conclusions:**

The overall quality of CPGs, as assessed by the four experts with the AGREE II instrument, was low. The highest scoring, best organized and most comprehensive and straightforward CPG was the one for chronic pancreatitis. The AGREE II instrument should be considered for use in Russia by guideline developers to assess existing CPGs and inform the creation of new guidelines.

## Introduction

Clinical practice guidelines (CPG) are a useful tool for evidence-based healthcare provision. The World Health Organization (WHO), governmental health agencies and civil society organizations at the country level work to introduce them into clinical practice. The main goal of CPGs is to provide to practicing physicians the tools for quality evidence-based health care [1]. However, in general, physician adherence to CPGs is low [2]. In Russian Federation, the majority of official guideline documents have been developed in the past two decades. However, health care professionals in Russia mostly use these guidelines to make insurance and hospital financing decisions [3, 4, 5], not to inform clinical decisions.

In some instances, conflicting CPGs for treating the same condition may be available, originating from different professional organizations. In these cases, physicians need to make an informed decision about which guidelines to use. The quality and reliability of CPGs is very important to physicians, as the health outcomes of their patients directly depend on CPGs implementation. In this study we examine the quality of Russian CPGs for various conditions affecting the hepatopancreatobilliary system. In particular, we analysed CPGs for acute cholangitis, acute cholecystitis, acute pancreatitis, chronic pancreatitis and cholelithiasis by using AGREE II assessment tool.

AGREE instrument is an internationally recognized, validated tool for guideline development and assessment. It was introduced in 2003, and later updated in 2009 to become AGREE II. It allows assessing methodological rigour and transparency in which a guideline was developed [1]. Ideally, AGREE II is to be used in the process of guideline development, as it provides a checklist for the most important issues that must be considered when developing any guideline universally. AGREE II consists of 6 domains with several questions each, which makes for a total of 23 items [1]. AGREE II is often used in the Western world, but it is rarely used in Russia for guideline assessment and has never been used for guideline development. The use and importance of the AGREE II instrument are further discussed in the Methods section. For the convenience of our experts, the team of Cochrane Russia translated AGREE II into Russian (S3 Text).

Pathologies of the hepatopancreatobiliary system present the most common disorders of the digestive tract, managed surgically [6]. Due to the complex anatomical structure and topography of this zone, severity of complications with high mortality rates and new costly technologies used for their management, hepatobiliary and pancreatic disorders increasingly present challenges to health systems [6]. In Russia, diseases of digestive system affect 5% of total population, or 66.1 per 100 thousand people and present the third most common cause for hospital admissions with consistent increase from year to year [7].

Acute cholangitis is a bacterial infection that causes inflammation and obstruction of the biliary tract, which, if left untreated, it is potentially lethal [8]. As the amount of bacteria in bile grows exponentially, the bacteria gets into the bloodstream, cause sepsis and, eventually, death [8]. In Russia acute suppurative cholangitis complicated up to 27% of patients with cholelithiasis [9]. The Russian Society of Surgeons created CPGs for treating acute cholangitis in 2016 [10]. A brief description of the content of this CPG can be found in S1 Text.

Acute cholecystitis is an inflammation of the gall bladder. It is caused mainly by gallstones blocking the cystic duct [11]. It is characterized by sharp pain in the upper right abdomen and may lead to gallbladder rupture if left untreated [12]. The Russian Society of Emergency Medicine published CPGs for treating acute cholecystitis in 2014 [13]. The Russian Society of Surgeons created national CPGs for treating acute cholecystitis in 2015 [14]. A brief description of the contents of these CPGs, along with the major differences between them, can be found in S1 Text.

Cholelithiasis is a condition where gallstones are present in the gallbladder. Gallstones may block the bile duct and cause severe dull pain to the patient [15]. The Russian Gastrointestinal Association created a set of CPGs on choleliathisis in 2015 [16]. A brief description of the content of this CPG can be found in S1 Text.

Acute pancreatitis happens when the pancreas quickly becomes inflamed and the patient experiences sharp pain in the abdomen [11, 17]. Its prevalence in Russia ranges from 32 to 389 per million population, with death rate ranging from 6 to 12 people per 100 [18]. The Russian Society of Surgeons, the Association of Hepatopancreatic Surgeons of CIS Countries, and the Russian Society of Emergency Medicine created a collective set of CPGs for treating acute pancreatitis in 2014 [19]. A brief description of the content of this CPG can be found in S1 Text.

On the other hand, chronic pancreatitis is a condition where inflammation of the pancreas recurs over many years [18]. In Russia chronic pancreatitis accounts for about five to 9% of all digestive diseases, considerably reducing patient quality of life [20]. The Russian Society of Surgeons and the Association of Hepatopancreatic Surgeons of CIS Countries created a collective set of CPGs for treating chronic pancreatitis in 2014 [21]. A brief description of the content of this CPG can be found in S1 Text.

Noteworthy is the recent consistent growth of benign and malignant pathology of pancreatic-biliary system with consequent growth of the prevalence of the syndrome of mechanical jaundice [20]. Despite the introduction of modern sophisticated diagnostic procedures and minimally invasive endoscopic and percutaneous trans-hepatic interventions, there are many complications with lethality rates reaching 15–30%. [22].

One of the best tools used to analyse CPGs is a questionnaire-based tool called Appraisal of Guidelines for Research & Evaluation (AGREE). Now AGREE II is available for use. The AGREE II tool provides an opportunity to analyse the guidelines based on six distinct domains. Those domains are *Scope and Purpose*, *Stakeholder Involvement*, *Rigour of Development*, *Clarity of Presentation*, *Applicability*, and *Editorial Independence* [23]. We asked four experts in gastrointestinal surgery to assess the identified guidelines using this tool.

### Aim

We aimed to analyse the quality of different Russian-produced CPGs available for surgical treatment of hepatic-pancreatic-biliary diseases (specifically acute cholangitis, acute cholecystitis, cholelithiasis, acute pancreatitis and chronic pancreatitis) in Russia.

## Methods

### Design

A descriptive study with qualitative and quantitative research methods.

### Eligibility criteria

We included CPGs on acute cholangitis, acute cholecystitis, acute pancreatitis, chronic pancreatitis and cholelithiasis published between 2013 and 2017 in Russian language that were meant for health care professionals, specialising in surgery, providing care for adult patients in a hospital setting. The CPGs had to be available online.

### Search methods for identification of guidelines

We searched for all relevant guidelines (CPGs) regardless of the level of their acceptance by either professional associations or health systems managerial bodies (approved / accepted for implementation, draft consultations / in development).

We used a PRISMA flow checklist to create the PRISMA diagrams for our literature searches (S1 Checklist). We conducted the primary search in October—December 2016 (S1 Fig), then repeated and expanded our search in April 2018, with the date of the last search 12th April 2018 (S2 Fig).

In 2016 we searched the following Russian databases:

- Federal Electronic Medical Library of the Ministry of Health of the Russian Federation (http://www.femb.ru/feml or http://feml.scsml.rssi.ru/feml)
- Websites of professional associations:
  - Russian Society of Surgeons (http://xn----9sbdbejx7bdduahou3a5d.xn--p1ai/)
  - Russian Society of Emergency Medicine (http://www.emergencyrus.ru/#/home/)
  - Russian Association of Gastroenterologists (http://www.gastro.ru)

In 2018 we repeated the search in the databases we searched in 2016 and added the following two sources to the list:

- eLIBRARY.RU (www.elibrary.ru);
- official collection of clinical practice guidelines of the Ministry of Health (http://cr.rosminzdrav.ru/#!/)

### Selection and analysis of CPGs with description of the databases and website

#### 1. Federal Electronic Medical Library of the Ministry of Health of the Russian Federation

Searched in 2016 and 2018. It was established by the Ministry of Health in 2012 on the basis the Central Scientific Medical Library of the First Moscow State Medical University named after I.M. Sechenov. It consists of seven databases, one of which is Clinical Guidelines (Protocols of treatment) with 1202 records on the 12th of April 2018. It is the primary electronic database for clinical practice guidelines. It operates in Russian only, allows simple and advanced search with the use of Boolean operators and filters for adults and children, and for the level of healthcare (emergency care, in-patient care, out-patient care and day-time in-patient care). All searches with results are stored in history. We used simple search terms, filtering for adults and not filtering for the level of care. Here we found four CPGs meeting our inclusion criteria (all approved):

- on acute cholecystitis—2 CPGs:
  - 2015 from the Russian Society of Surgeons, and
  - 2014 from the Russian Society of Emergency Medicine
- on acute cholangitis—no results
- on acute pancreatitis– 1 and the same CPG with 2 records:
  - 2014 jointly from the Russian Society of Surgeons, the Association of Hepatopancreatic Surgeons of CIS Countries, and the Russian Society of Emergency Medicine
  - 2014 from the Russian Society of Emergency Medicine
- on chronic pancreatitis—1 CPG jointly from the Russian Society of Surgeons, the Association of Hepatopancreatic Surgeons of CIS Countries, 2014
- on cholelithiasis—no results

#### 2. The website of the Russian Society of Surgeons

Searched in 2016 and 2018. It has a series of special pages for CPGs: approved for use Clinical Guidelines grouped in 8 sections, including abdominal surgery and emergency abdominal surgery; draft guidelines for comments and suggestions grouped in 4 sections; drafts to be approved at the 12th National meeting of the surgeons of Russia (acute appendicitis); and methodology papers on the process of guideline development. The website uses only Russian language; allows for eye screening of guidelines. Here we found four CPGs meeting our inclusion criteria:

- approved CPGs on acute cholecystitis (2015),
- approved CPGs on acute pancreatitis (2014),
- approved CPGs on chronic pancreatitis (2014), and
- a draft of CPGs on acute cholangitis (2016).

#### 3. The website of the Russian Society of Emergency Medicine

Searched in 2016 and 2018. It does not contain any clinical guidelines. It provides the website visitors with description of organisational structure and leadership of the Society, relevant governmental decrees and orders, information on events; it also provides platform for questions and answers, and the news. The website uses only Russian language. The CPGs, developed and approved by this Society are located on the Federal Electronic Medical Library of the Ministry of Health of the Russian Federation and on the website of the Russian Society of Surgeons.

#### 4. The website of the Russian Association of Gastroenterologists

Searched in 2016 and 2018. It consists of five major pages, delivering information on upcoming conferences, the association’s clinical practice guidelines, about the association, announcements and specifics for continuing medical education. The website uses only Russian language. There are 24 CPGs available for cursory screening. We identified two CPGs, meeting our inclusion criteria:

- on cholelithiasis (2015) and
- on chronic pancreatitis (produced in 2013, published 2014 identified in both searches of 2016 and 2018).

#### 5. The official collection of clinical practice guidelines of the Ministry of Health (MoH)

Rubriсator–Рубрикатор, searched in 2018. It contains searchable collection of CPGs, grouped according to the International Classification of Diseases 10. It was launched in October—November 2017, establishing the system of National Clinical Guidelines. This database operates in Russian only, allows simple search by terms, registered number of a CPG, year of publication, ICD position, status (approved in action, draft in development and outdated) with filters for adults and children. Here we found three CPGs meeting our inclusion criteria (all approved):

- on acute cholecystitis (2015 from the Russian Society of Surgeons), the same as 2014, formatted to a new style
- on acute pancreatitis (2014 jointly from the Russian Society of Surgeons and the Association of Hepatopancreatic Surgeons of CIS Countries and the Russian Society of Emergency Medicine), and
- on chronic pancreatitis (2016 from Russian Association of Gastroenterologists).

#### 6. eLIBRARY.RU

It is the largest Russian electronic database of scientific publications; it is searchable. The database is integrated with the Russian Scientific Citation Index (RINC), which is a free publicly available tool for measuring publication activity of individual researchers and organizations, commissioned by the Ministry of Education and Science of the Russian Federation. The eLIBRARY.RU and RINC have been developed and supported by the Scientific Electronic Library Company. The platform eLIBRARY.RU was created in 1999 on the initiative of the Russian Foundation for Basic Research to provide Russian scientists with electronic access to leading foreign scientific publications. Since 2005, eLIBRARY.RU has started working with Russian-language publications and is now the leading electronic database of scientific periodicals in Russian in the world. Abstracts and full texts of more than 26 million research papers and publications, including electronic versions of more than 5300 Russian scientific and technical journals are available at eLIBRARY.RU. The total number of registered institutional users (organizations) is more than 2800. The system registered 1.7 million individual users from 125 countries. Annually readers receive from the library more than 12 million full-text papers and view over 90 million annotations. More than 4,500 Russian scientific journals are placed in free public access. For access to other publications, it is possible to subscribe or order individual publications. eLIBRARY.RU operates in Russian only, though it contains some publications in other languages, including English. We ran a series of searches by surgical condition / pathology of interest, which yielded finally 2 potential records, which were excluded after assessing full texts for eligibility (S1 Table).

### eLIBRARY search strategy

This strategy is based on search specifics of the database: search by term morphology, meaning by the roots of the words typed in as full words (с учетом морфологии); search by combination of terms, working as Boolean operator OR; searching within results of the previous search working as Boolean operator AND; combining searches in the automated way to remove duplicates.

1. acute cholecystitis AND clinical guidelines (острый холецистит И клинические рекомендации)
2. acute cholangitis AND clinical guidelines (острый холангит И клинические рекомендации)
3. acute pancreatitis AND clinical guidelines (острый панкреатит И клинические рекомендации)
4. chronic pancreatitis AND clinical guidelines (хронический панкреатит И клинические рекомендации)
5. cholelithiasis AND clinical guidelines (желчнокаменная болезнь И клинические рекомендации)

We identified a total of 6 CPGs (Table 1).

**Table 1:** Clinical practice guidelines assessed with AGREE II instrument.

Condition	Organization	Year	Organization abbreviation
Acute cholangitis	Russian Society of Surgeons	2016	RSS
Acute cholecystitis	Russian Society of Surgeons	2015	RSS
Acute cholecystitis	Russian Society of Emergency Medicine	2014	RSEM
Cholelithiasis	Russian Gastrointestinal Society	2015	RSG
Acute pancreatitis	Russian Society of Surgeons, Association of Hepatopancreatic Surgeons of CIS Countries, Russian Society of Emergency Medicine	2014	RSS, AHS of CIS, RSEM
Chronic pancreatitis	Russian Society of Surgeons and Association of Hepatopancreatic Surgeons of CIS Countries	2014	RSS and AHS of CIS

The four experts performed the assessment independently of each other. Each expert had a different specialization in the field of abdominal surgery (S1 Appendix), and thus presented a different approach to assessing the guidelines, which was most useful in obtaining a well-rounded, balanced analysis of CPGs. We equipped all four experts with the AGREE II instrument and with detailed information on how to use it. But we did not provide any formal training on the AGREE instrument.

The AGREE II is a questionnaire designed to assess the quality of health care guidelines and to be used as a tool to develop clinical practice guidelines [23]. AGREE II has been available in English since 2010; the team of Cochrane Russia translated it into the Russian language (S3 Text).

AGREE II includes 23 questions in 6 different domains that address different aspects of guidelines. Those domains are *Scope and Purpose* (3 questions), *Stakeholder Involvement* (3 questions), *Rigour of Development* (8 questions), *Clarity of Presentation* (3 questions), *Applicability* (4 questions), and *Editorial Independence* (4 questions) [23]. For each question, the expert is asked to provide a numerical score between 1 and 7, 1 being the minimum score and 7 being the maximum score. Each question is presented as a statement and the experts should answer the questions based on how much they agree with the statement (1 –strongly disagree, 7 –strongly agree). Each CPG receives a raw score between 23 and 161 points from a single expert. It is recommended that at least two experts analyse the same guidelines using the AGREE II tool. Additionally, experts may leave comments for each question and state whether they would recommend the use of the guideline at the end of the questionnaire.

Next, derived data is calculated by compiling the scores of all experts for each domain and scaling them as a percentage of a maximum possible score for that specific domain. That is done by using the formula (Eq 1):

$$\frac{Obtained\;score-Minimum\;possible\;score}{Maximum\;possible\;score\;–Minimum\;possible\;score}\times 100\%$$ (1)

Table 2 below provides a sample set of data for *Scope and Purpose* domain for acute cholangitis CPG by the Russian Society of Surgeons (RSS), in which we show sample calculations to illustrate how the AGREE II scoring was used in our study:

**Table 2:** Sample data set for the domain *Scope and Purpose*.

	E1	E2	E3	E4	Total (n)	Total %
**SCOPE AND PURPOSE**	**19**	**9**	**5**	**15**	**48**	**50**
The overall objective(s) of the guideline is (are) specifically described.	7	4	1	4	16	
The health question(s) covered by the guideline is (are) specifically described.	5	3	2	4	14	
The population (patients, public, etc.) to whom the guideline is meant to apply is specifically described.	7	2	2	7	18	

The total obtained score is calculated by summing the individual scores of all experts (Eq 2):

19+9+5+15=48 (2)

The minimum possible score is calculated by attributing 1 to each question, as the minimum possible score is 1 for each question and multiplying it by the number of experts (Eq 3):

(1+1+1)×4=12 (3)

The maximum possible score is calculated by attributing 7 to each question, as the maximum possible score is 7 for each question and multiplying it by the number of experts (Eq 4):

(7+7+7)×4=84 (4)

The original formula is used to calculate the domain score (Eq 5):

$$\frac{Obtained\;score\;-\;Minimum\;possible\;score}{Maximum\;possible\;score\;–\;Minimum\;possible\;score}\times 100\%\;=\;\frac{48-12}{84-12}\times 100\%=50\%$$ (5)

We performed this calculation in Microsoft Excel. We created a separate data sheet for each CPG and another sheet—for the comparison of all guidelines. First, we created a table for each CPG with all 23 questions organized in the 6 domains. Each table included the data from four experts. We added the scores for each question from the four experts. Next, we calculated minimum and maximum scores for each domain. Then, we calculated the difference between the maximum and the minimum score for each domain. We used the above formula to calculate a domain score for each domain and the total sum of assessment for each CPG. Since we analyzed two CPGs for treatment of acute cholecystitis, we also created a separate comparison table for these two CPGs. Finally, we created a comparison table for all CPGs and made a bar graph to visualize the difference in total sum of assessment between all the CPGs studied.

## Results

### Domain scores

The domain scores differed greatly between different CPGs. The domains *Scope and Purpose*, and *Clarity of Presentation* consistently received high scores across all CPGs. However, the domains *Applicability* and *Editorial Independence* consistently received low scores.

The domain *Scope and Purpose* deals with the main objective of CPGs, the health question and the population to whom the guideline is meant to apply [23]. This domain includes 3 items (items 1–3). The range of scores for *Scope and Purpose* was 35%–100% (S9 Table). Most CPGs received high scores for this domain (50%–100%), while two CPGs received low scores of 35% and 36%. The CPG for treating acute cholecystitis by the Russian Society of Surgeons obtained the lowest score for this domain (S5 Table). The experts attributed it to the guidelines having a vague and not clearly defined objective. They provided similar critique about the CPG for treating acute pancreatitis (S7 Table). The CPG for treating chronic pancreatitis obtained the highest score for *Scope and Purpose*s as the population was clearly described and the purpose was well defined (S8 Table).

The domain *Stakeholder Involvement* is focused on assessing whether all relevant clinical professionals participated in the development of the CPG and whether the target audience who would use the CPG is specified [23]. This domain includes three items (items 3–6). The range of scores was 24%–76% (S9 Table). The CPG for treating acute cholangitis received the lowest score and the CPG for treating acute cholecystitis by the Russian Society of Emergency Medicine received the highest score (S2 and S5 Tables).

The experts assigned such a low score (24%) to the CPG for treating acute cholangitis for the domain *Stakeholder Involvement* as it did not include any specifications about the target audience and only surgical specialists participated in the development of the guidelines. All experts pointed out that clinical pharmacologists, general physicians, epidemiologists, and medical statisticians should have been included in the development of CPG for treating acute cholangitis.

The CPG for treating acute cholecystitis by the Russian Society of Emergency Medicine received the highest score (76%) for the domain *Stakeholder Involvement* (S5 Table). The experts were unanimous in their comments regarding the fact that more stakeholders could have been involved in the development of these guidelines, but overall the variety of specialists involved in the development of these guidelines was better than in all other CPGs. A similar score (74%) for the domain *Stakeholder Involvement* was obtained by the CPG for treating chronic pancreatitis (S8 Table). That set of CPGs received similar comments from the experts.

The domain *Rigour of Development* deals with the actual methods used to compose the CPGs [23]. It is the largest domain in the AGREE II and consists of eight items (items 7–14). The range of scores for this domain was 9%–81% (S9 Table). *Rigour and Development* had the largest variation between the highest and the lowest scores, as it is the biggest and arguably the most important domain in the AGREE II. The scores for all CPGs varied greatly.

The lowest score for the domain *Rigour and Development* was obtained by the CPG for treating acute cholangitis (S2 Table). Experts supported their decision of giving this CPG such a low score by providing extensive comments. They pointed out that the CPG lacked a procedure of evidence search, strengths and limitations of this evidence and most other items of this domain. The only item in the *Rigour and Development* that received a score above 1 from three of the experts was the item 10, which deals with methods for creating the recommendations.

The highest score (81%) for the domain *Rigour and Development* was obtained by the CPG for treating chronic pancreatitis (S8 Table). Even though the experts gave this CPG a high score, they left many comments for this domain. Most experts were unanimous in saying that the CPG for treating chronic pancreatitis did not include a method for reviewing the strengths and limitations of the evidence (such as GRADE), or a Delphi method for formulating the recommendations.

The fourth domain is called *Clarity of Presentation* and it consists of three items (items 15–17). *Clarity of presentation* is focused on the way the CPG is written, specifically its language, format, and structure [23]. This domain consistently received a high score in each of the CPGs (47%–81%) (S9 Table). The CPG for treating acute cholangitis received the lowest score (47%; S2 Table). The experts attributed it to the CPG missing crucial information, such as practical clinical questions and hiding the main recommendations in the body text, instead of pointing it out in the form of a table. The highest scores for this domain were achieved by the CPG for treating cholelithiasis (81%) and chronic pancreatitis (80%). The experts were unanimous in their comments, stating that the recommendations were unambiguous and clear (S6 and S8 Tables).

The next domain is called *Applicability* and it involves four items (items 18–21). *Applicability* mostly assesses the way that the CPG can be used in the society, including crucial factors such as assessment of cost, barriers and facilitators of implementation, as well as in possible tactics for uptake of the recommendations [23]. Overall, *Applicability* received a low score from the experts throughout all CPGs (8%–55%) (S9 Table).

The lowest score (8%) for the *Applicability* domain was received by the CPG for treating acute cholangitis (S2 Table). The experts simply stated that there was not enough information about the implementation factors in the CPG. On the other hand, the CPG for treating cholelithiasis received the highest score for the domain *Applicability*, even though the experts indicated that they could not locate some of the information needed for this domain, such as the criteria for auditing and the possible facilitators and barriers to the application of the CPG (S6 Table).

The last domain in the AGREE II is called *Editorial Independence* and it consists of two items (items 22–23). This domain score reflects whether the authors could have any conflict of interest in the matter of creating the CPG [23]. This domain received the lowest scores from all the experts. The range of the scores was 6%–25% (S9 Table).

The lowest score of 6% was obtained by the CPG for treating acute pancreatitis (S7 Table). All experts gave it the lowest rating (1 point) by stating that no information about the conflict of interest was provided, but Expert 4 gave this CPG 4 points for the item 22, which assesses whether the funding influenced the recommendations. The Expert 4 said that he simply did not know the answer to that question, and gave the item 4 points out of 7 possible points, which led to the CPG receiving 6% overall score for the domain *Editorial Independence*, instead of 0%.

The highest score (even though it was considered low as a separate score) was received by the CPG for treating chronic pancreatitis and the CPG for treating acute cholecystitis by the Russian Society of Emergency Medicine (both received 25%; S4 and S8 Tables). The experts provided no particular comments for their rating. Experts 1, 2 and 3 gave the lowest possible rating to this CPG (1 point), but expert 4 gave it the highest rating without any explanation, which was the reason that the CPGs for treating chronic pancreatitis and acute cholecystitis have a 25% rating.

### Total sum of assessment scores

The total sum of scores, or the overall calculation was performed for each of the CPGs. Their comparison can be viewed in S9 Table. It was used to assess the overall quality of the CPGs. Overall, the highest score (70%) was received by the CPG for treating chronic pancreatitis (S8 Table). The experts only gave low scores to the domains *Applicability* and *Editorial Independence*, but those domains received low scores in all CPGs.

The lowest total sum score was obtained by the CPG for treating acute cholangitis (22%; S2 Table). It received scores of 50% or less for all domains. The authors attributed this to the CPG still being under development and suggested that many changes should be made before the CPG for treating acute cholangitis should be introduced into clinical practice.

Two different CPGs for treating acute cholecystitis were analysed (S3 and S4 Tables). The CPG for treating acute cholecystitis by the Russian Society of Emergency Medicine received a total sum score of 41%, while the CPG for treating acute cholecystitis by the Russian Society of Surgeons received a total sum score of 32% (S5 Table). Even though both scores were low, the experts attributed higher quality recommendations to the CPG from the Russian Society of Emergency Medicine.

## Discussion

Evidence-based medicine has become the mainstream and key to the development of clinical medicine and health care systems over the last thirty years [24]. Different teams developed multiple CPGs over time and there are no set standards as to which guidelines to use, so physicians have to use their best judgment in deciding which CPGs to implement. Although there are many papers describing how to determine whether a certain CPG is of good quality, qualitative and quantitative data gathered with AGREE II instrument currently is the best validated tool for a physician or a health setting / system to decide whether to implement guidelines or not [23]. CPG is a relatively new concept for the Russian health community, thus, difficulties in creating high quality CPGs still exist. There are no yet fully agreed upon and accepted official standards on how the CPGs are to be formulated and formatted or what information must be included. Our findings of low overall scores for all studied CPGs confirm this.

We found the combination of two problems—variability in assessment scores across the people using the AGREE tool and variability in quality of the assessed CPGs. We think that there are many reasons for this variability, including the following.

As the concept of CPGs is relatively new, most organizations that create CPGs get formal approval of a CPG at their conferences or other important professional meetings, where many experts in the field are present. Every CPG is different in terms of its structure, content organization, use of evidence, etc. Often, it is unclear who the authors are or what their clinical specialty is, as only their degrees, such as PhD or DSc are mentioned.

Two CPGs (chronic pancreatitis and acute pancreatitis) were developed by a coalition of organizations, while all other CPGs were created by single organizations (Table 1). Interestingly, the CPG for treating chronic pancreatitis created by the coalition of the Russian Society of Surgeons and the Association of Hepatopancreatic Surgeons of CIS Countries received the highest total assessment score among all studied CPGs (70%), while the CPG for treating acute pancreatitis by the same organization in partnership with the Russian Society of Emergency Medicine received the second lowest total assessment score (24%) (S7 and S8 Tables). The discrepancy of scores is especially noteworthy as the acute pancreatitis CPG was created by three organizations, and the one for chronic pancreatitis was developed by the coalition of two of those organizations.

Two CPGs for treating acute cholecystitis created by two different organizations were assessed. Their total assessment scores were both low, but the CPG by the Russian Society of Emergency Medicine had a higher score than the one created by the Russia Society of Surgeons (S5 Table). Noteworthy is the lack of consistency in quality even among CPGs created by the same organization, specifically the Russian Society of Surgeons.

The quality of some CPGs should indeed be questioned due to complete absence of in-text citations. Only two of six CPGs (for acute cholangitis, and for chronic pancreatitis) consistently cited references throughout the text; the CPG for cholelithiasis only referred to literature citations in the history section. Also, the majority of CPGs except for acute pancreatitis and for acute cholecystitis by the Russian Society of Emergency medicine, used mostly sources published in English and not in Russian. This may be another reason for variable quality of CPGs as assessed by the experts, as many recommendations that are easily implemented in Western countries may not be practical in some regions of Russia due to technological and financial constraints. We did not find any interrelation between the quality of a CPG and the provided references.

Overall, the scores in each domain varied greatly from one expert to the other. One of the reasons, why the answers of the experts differed so much, was that each of them specialized in a different aspect of abdominal surgery, ranging from endoscopic and endocrine surgery to emergency general surgery.

We think that one more reason for these variations might be that Russian medical education is very different from that of Western countries. Our healthcare professionals are not trained to critically appraise literature, to apply methodology concepts, or to assess any health policy documents. From our experts’ comments we notice that they may be biased towards certain parts of the CPGs depending on whether they agree with the proposed treatment option or not, rather than assessing the methodological aspects of the CPGs per se. In addition to this, some of the CPGs more closely resemble reviews of Western (mostly British or American) CPGs rather than an analysis of evidence in creating CPGs appropriate for Russia specifically.

It is interesting to note that Expert 4 gave very high ratings to most CPGs, while the other three experts gave consistently low and similar scores and comments to all CPGs. This could possibly be attributed to the fact that the first three experts were located in one city (Kaliningrad), while expert 4 was located in Kazan. Experts 1, 2, and 3 could have communicated among themselves or had been influenced by the same factors, while expert 4 was not influenced by those factors. Also, Kaliningrad is the only city in Russia that is located away from the rest of the country. It is located in Europe, so the first three experts may have had more exposure to Western methodology assessment techniques than the expert in Kazan did. It is also a possibility that the instructions for the use of AGREE II were better explained to the first three experts, as they were handed the CPGs in person, while expert 4 only received them over e-mail with little guidance on how to analyse them.

None of the experts received practical training on how to use the AGREE II tool. This explains the fact that many times experts gave a low sum score to a particular CPG, but rated it 7/7 for the overall quality (last question on the AGREE II questionnaire, not used in the calculation of total points) and said they would recommend the use of guidelines with some changes. Often, the experts questioned the validity of the guidelines due to absence of factors that they deemed important, but still said that they would use those CPGs. The AGREE II instrument is not meant to test the validity or reliability of guidelines, simply the methods of their creation and the potential to be implemented in the society [25].

We also assessed the Russian CPGs independently to see whether our results would be different from those given by the experts (S10–S17 Tables). Each author analyzed the CPGs with the AGREE II tool separately and independently. We did not refer to each other’s scores for comparison. After each of the authors scored the CPGs and provided comments, we followed the same protocol as described in the Methods section to calculate domain scores and total sum of assessment scores for each CPG. We also made summarizing tables (S9 and S17 Tables) and bar graphs to easily visualize the difference in scores between the experts and the authors.

**Fig 1:**
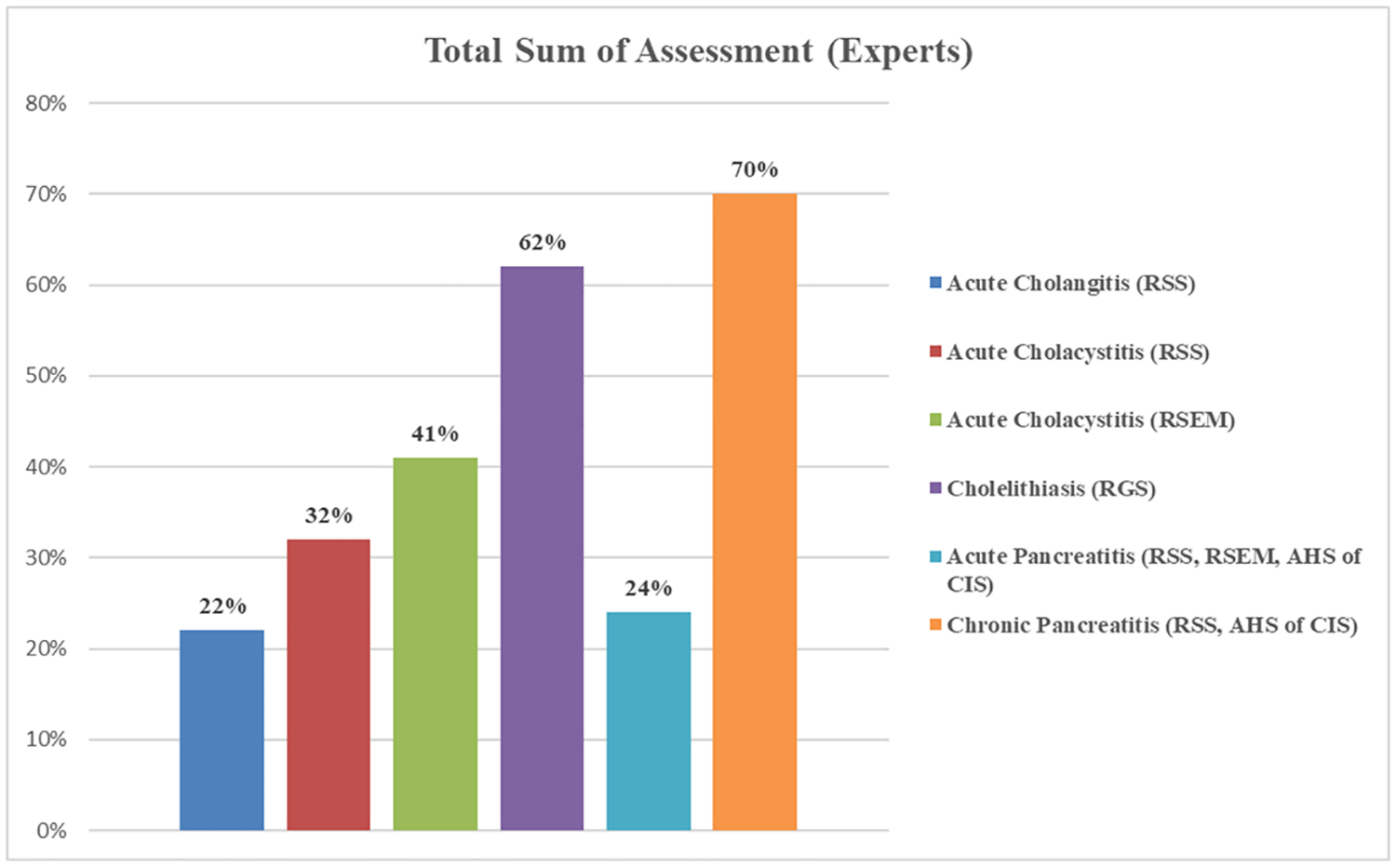
Total sum of assessment scores of Russian CPGs from the experts.

**Fig 2:**
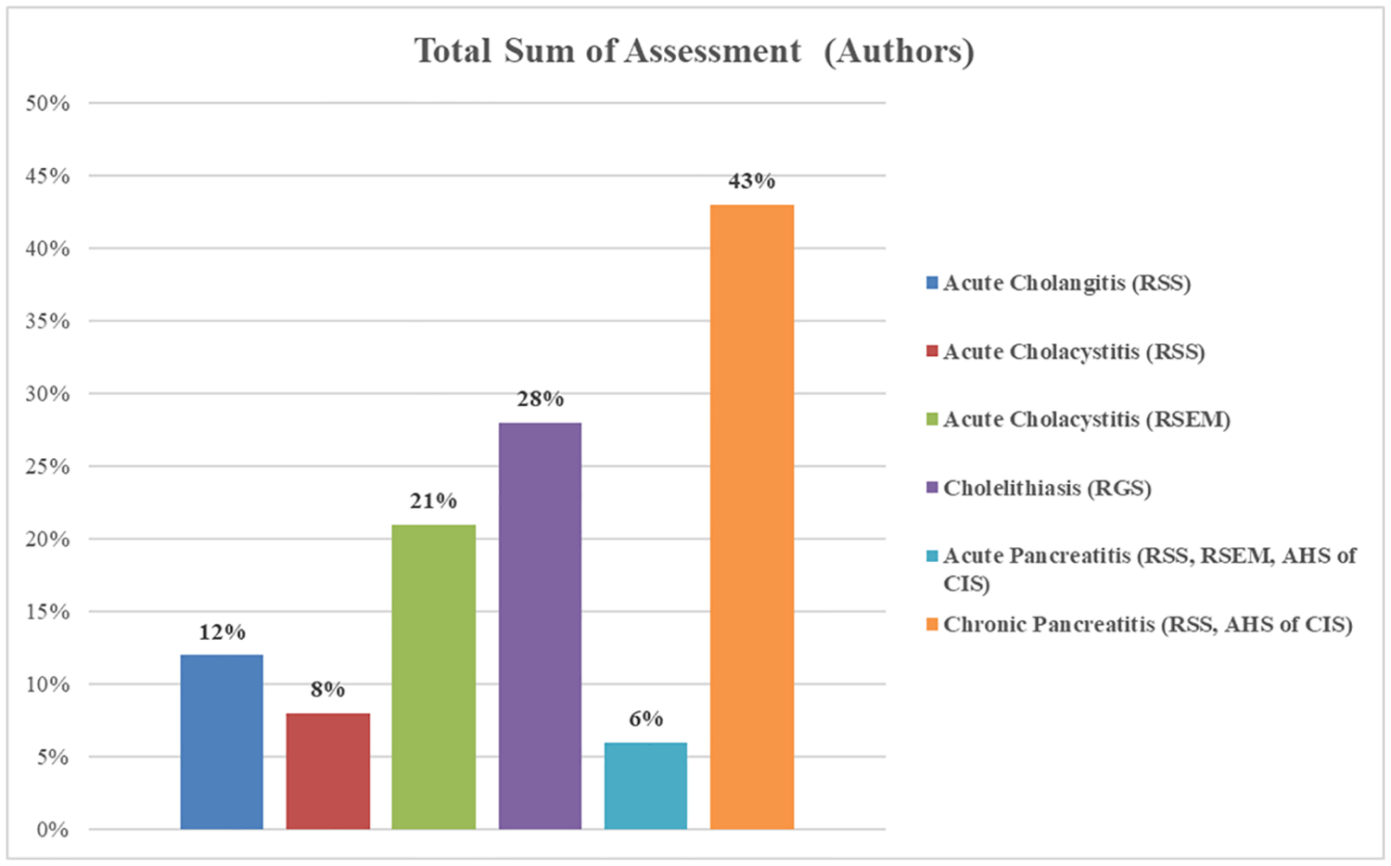
Total sum of assessment scores of Russian CPGs from the authors.

Interestingly, as the figures below show, we gave overall lower total sum of assessment scores to all guidelines, yet the general trend of which CPGs got higher / lower scores was similar to that of the experts (Figs 1 and 2). The abbreviations in those figures correlate to those in Table 1. Our scores were about 1.5 to 4 times lower than those of the experts. We made every effort to only analyse methodological quality of guidelines and the possibility of being practically implemented in Russia, as intended by AGREE instrument [25]. We gave a 0% score to all CPGs for the last domain, *Editorial Independence*, as not a single CPG specified any funding or competing interests in their text. It is possible that our experts were more familiar with the authoring organizations of the CPGs and thus assessed the last domain based on their prior knowledge of possible funding and competing interests. The detailed scores for each domain of each CPG can be found in S10–S16 Tables.

Even though there was a large difference in the scores, we rated the CPG for treating chronic pancreatitis as the highest quality one, just as the experts did. Thus we think that these our findings would not change much if some formal cursory training for experts was performed. In fact, we plan for the trainings with pre- and post-training assessment, which we will hopefully be able to perform in the design of a randomized controlled trial (AGREE assessments by experts after training versus no training). However, by the time we will be able to have training performed there will be new editions of the CPGs, which we hope will be better due to the current momentum in the country with high governmental attention to guideline process, including our humble contribution with this manuscript.

The appreciation of the value of evidence-based guidelines by the Russian health system leaders and the high level governmental leadership has been growing recently, with the launch of the official collection of clinical practice guidelines of the Ministry of Health of the Russian Federation (Rubriсator–Рубрикатор) in late 2017 thus establishing the system of the National Clinical Guidelines. Our repeated searches of April 2018 showed that this has not yet solved the problem of multiple guideline documents developed and endorsed by various professional organisations or health policy makers. We hypothesize that our results of AGREE II assessment of this small collection of guidelines may be generalised to other clinical fields. However, this needs to be studied in a special project of applying the AGREE II tool to various sets of guidelines.

The AGREE II instrument is rarely used in Russia to assess guidelines, and there is no information whether any of the developers of the CPGs, assessed in this study, used AGREE II to develop them. The Russian situation with CPGs’ development seems to be typical for other upper middle-income countries, like China [26], or Turkey [27], as assessed with AGREE II instrument. The current Russian situation calls for urgent energetic action for guideline assessment, development and application.

We plan to undertake a follow-up study in two-three years’ time to determine whether the overall quality of Russian CPGs for treating hepatopancreatobiliary pathologies improved.

### Study limitations

Only CPGs available online were analysed. Institutional or any other CPGs were not analysed. The experts received no formal training with the AGREE II instrument.

## Conclusion

The overall quality of the CPGs was low as the total sum score ranged between 22% and 70% (S9 Table). All CPGs needed improvements, especially in the *Rigour of Development* domain. The highest scoring domain of the AGREE II was Clarity of Presentation among all CPGs and the lowest scoring domains were *Applicability* and *Editorial Independence*. The highest scoring, best organized and most comprehensive and straightforward CPG was the one for chronic pancreatitis.

These findings and the limitations of this research are important to the Russian health community as a whole, as they show that Russian medical professionals are not skilled enough in the development of Evidence-Based Clinical Practice Guidelines or use of the AGREE II instrument. The results also show that the AGREE II instrument can be used efficiently in assessing existing Russian CPGs and producing better quality clinical recommendations in the future.

## Supporting information

S1 Text Descriptions of CPGs. (DOCX)

S2 Text Cochrane Russia funding statement. (PDF)

S3 Text AGREE II instrument translation into Russian. (DOCX)

S1 Checklist PRISMA 2009 checklist. (DOC)

S1 Fig PRISMA diagram 2016. (PDF)

S2 Fig PRISMA diagram 2018. (PDF)

S1 Appendix Expert panel description. (DOCX)

S1 Table Reasons for exclusion. (DOCX)

S2 Table Assessment of CPG for treating acute cholangitis with AGREE II. (XLSX)

S3 Table Assessment of a CPG for treating acute cholecystitis with AGREE II (by the Russian Society of Surgeons). (XLSX)

S4 Table Assessment of a CPG for treating acute cholecystitis with AGREE II (by the Russian Society of Emergency Medicine). (XLSX)

S5 Table Comparison of the assessment of two CPGs for treating acute cholecystitis with AGREE II. (XLSX)

S6 Table Assessment of CPG for treating cholelithiasis with AGREE II. (XLSX)

S7 Table Assessment of CPG for treating acute pancreatitis with AGREE II. (XLSX)

S8 Table Assessment of CPG for treating chronic pancreatitis with AGREE II. (XLSX)

S9 Table Total sum of assessment for all CPGs with AGREE II. (XLSX)

S10 Table Authors’ assessment of CPG for treating acute cholangitis with AGREE II. (XLSX)

S11 Table Authors’ assessment of a CPG for treating acute cholecystitis with AGREE II (by the Russian Society of Surgeons). (XLSX)

S12 Table Authors’ assessment of a CPG for treating acute cholecystitis with AGREE II (by the Russian Society of Emergency Medicine). (XLSX)

S13 Table Authors’ comparison of the assessment of two CPGs for treating acute cholecystitis with AGREE II. (XLSX)

S14 Table Authors’ assessment of CPG for treating cholelithiasis with AGREE II. (XLSX)

S15 Table Authors assessment of CPG for treating acute pancreatitis with AGREE II. (XLSX)

S16 Table Authors’ assessment of CPG for treating chronic pancreatitis with AGREE II. (XLSX)

S17 Table Authors’ total sum of assessment for all CPGs with AGREE II. (XLSX)
