# Supplementary material for: Assessing methodological quality of Russian clinical practice guidelines and introducing AGREE II instrument in Russia
Source: PLoS One. 2018 Sep 11;13(9):e0203328. doi: 10.1371/journal.pone.0203328 (PMC6133363; doi:10.1371/journal.pone.0203328)
Supplement: S1 Text — (DOCX) [file pone.0203328.s001.docx]

**S1 Text. Description of CPGs for hepatopancreatobiliary pathologies**

**Acute cholangitis**

The Russian Society of Surgeons created CPGs for treating acute cholangitis in 2016 [10]. It is 24 pages long (1.5-line spacing), which includes 83 references. From those references, 10 are in Russian and 73 are in English. The references are consistently cited in the body of the text. The words “recommended” (рекомендуется) and “not recommended” (не рекомендуется) are conveniently highlighted throughout the text. It does not have a title page and the authors are not listed. It just states that this is a document by the Russian Society of Surgeons and presents an ICD-10 code for acute cholangitis. The CPG begins with a brief explanation of what cholangitis is, how it is normally treated, and what the latest developments in treatment are.

The rest of the CPG is divided into 8 sections and the recommendations are made within those sections. The sections are: *diagnostic criteria* (which includes a table with the three grades of acute cholangitis); *examples of formulating a detailed diagnosis*; *the main methods of treatment* (which includes a descriptive table with details for each grade of cholangitis and a treatment checklist developed by a Russian scientist); *indications and methods of drainage of bile ducts*; *surgical bile drainage*; *anaesthesiology support of surgery*; *postoperative period*; and *antibacterial therapy*. The evidence levels A to D are consistently used and bolded throughout the text, but there is no reference as to what those levels symbolize.

**Acute cholecystitis 1**

The Russian Society of Surgeons created a national CPG for treating acute cholecystitis in 2015 [14]. It is 20 pages long (1.15-line spacing), which includes the title page and 130 references. From those references, 15 are in Russian and 115 are in English. There are no in-text citations or any mention of the references. The CPG begins with a title page, which proclaims this CPG as national and states that it was developed at the XII meeting of Russian surgeons in Rostov-on-Don. The next page begins with the list of authors, their affiliated cities and academic level (professor, associate professor, or PhD). There are 7 authors. Next, there is a table with the levels of evidence according to the Oxford Centre for Evidence-based Medicine. References to that table and the levels of evidence are made consistently throughout the text. After that there is a one sentence description of acute cholecystitis and 5 ICD-10 codes related to cholecystitis.

The rest of the CPG is divided into 8 sections and the recommendations are made within those sections. The sections are: *epidemiology*; *aetiology and pathogenesis*; *classification* (which includes a table on differential types of acute cholecystitis); *diagnosis* (which includes local and systemic signs of gallbladder inflammation, visual and differential methods of diagnosis, and 3 grades of severity of acute cholecystitis); *treatment* (which includes the aim of treatment, hospitalization, conservative and surgical treatment, timeframe of surgical treatment, surgery options and surgical treatment with accompanying diseases and complications, complications with obstructive jaundice, and treatment strategy); *antibacterial prophylaxis and drainage of sub-hepatic space*; *complications and their prevention*; and *prognosis*.

**Acute cholecystitis 2**

The Russian Society of Emergency Medicine published CPGs for treating acute cholecystitis in 2014 [13]. It is 18 pages long (1.0-line spacing), which includes the title page, 34 references, and an appendix with two tables. From those references, 19 are in Russian and 15 are in English. There are no in-text citations or any mention of the references. The CPG begins with a title page, which states that this CPG is designed specifically for emergency treatment of acute cholecystitis. The Russian Society of Emergency Medicine approved this CPG at their 2014 meeting. The next page begins with the list of authors, their affiliated institutions and hospitals, academic level (professor, or PhD), and medical specialty (emergency surgery, endoscopy, oncology). There are 7 authors. Next, there is a brief description of acute cholecystitis, specifically acute calculous cholecystitis, some epidemiology statistics, and the goals of the CPG. Afterwards, 8 ICD-10 codes related to cholecystitis are mentioned and a similar, but expanded classification for acute cholecystitis is provided, as in the previous CPG. The rest of the CPG is divided into two major sections – *emergency medical care for acute cholecystitis at the prehospital stage* and *emergency medical care for acute cholecystitis at the hospital stage in the inpatient ward*. Both of those sections have multiple subsections. All sections and subsections are conveniently numbered and bolded.

The first section, *Emergency medical care for acute cholecystitis at the prehospital stage* consists of 4 subsections. They are: *relevant medical history taking*; *physical exam* (which includes general symptoms for acute cholecystitis and symptoms for cholecystitis with perforation, cholangitis, and hepatitis complications); *differentiating acute cholecystitis from other pathologies*; and *pre-hospital treatment*.

The second section, *Emergency medical care for acute cholecystitis at the hospital stage in the inpatient ward* consists of 6 subsections. Each of the subsections has multiple sub-subsections. The first subsection is *Acute uncomplicated calculous cholecystitis*. It has 9 sub-subsections. They are: *general symptoms*; *physical exam*; *ultrasound*; *differential diagnosis*; *clinical examinations* (which includes required laboratory, instrumental, and additional examinations in case of comorbidities); *diagnosis criteria* (which includes a table with different criteria for catarrhal and destructive cholecystitis); *treatment strategy*; *conservative therapy*; and *indications for surgery*. The second subsection is called *Acute calculous cholecystitis complicated with choledocholithiasis with or without obstructive jaundice*. It has 6 sub-subsections. They are: *diagnosis of acute calculous cholecystitis complicated with choledocholithiasis*; *required laboratory examinations*; *required instrumental methods of examination*; *treatment strategy*; *conservative therapy*; and *indications for surgery*. The third subsection is *Acute cholecystitis complicated with peritonitis*. It has 3 sub-subsections. They are: *preoperative examination* (which includes a descriptive table with diagnosis, monitoring, required manipulations, therapy options, and infusion therapy); *therapy strategy for acute cholecystitis complicated with peritonitis*; and *indications for surgery* (which includes complications with limited and diffused peritonitis). The fourth subsection is *Acute cholecystitis complicated with formation of dense infiltrate*. It has 6 subsections. They are: *main symptoms of dense perivesical infiltrate*; *examination*; *differential diagnosis*; *patient management strategy* (which includes a descriptive table with options for infiltrate with and without abscessing); *treatment strategy*; and *indications for surgery and surgical method*. The fifth subsection is Acute cholecystitis with acute cholangitis. It has 3 sub-subsections. They are: *main symptoms*, *examination*, and *treatment strategy*. The sixth and last subsection *Acute cholecystitis complicated with biliary pancreatitis* has 3 sub-subsections. They are: *main symptoms*; *diagnosis of severe pancreatitis*; and *treatment strategy* (which includes conservative therapy and patient management strategy).

At the end of the CPG is a section titled *What must not be done*. It includes things that must not be done at both pre-hospital and hospital stages. This part is the first one that incorporates words such as “not recommended”. After the references there is an appendix. It consists of 2 tables, one with levels of evidence (1++ through 4) and one with strength of recommendation levels (A through D). The source of either table is not cited. The values in those tables are used consistently throughout the CPG.

**Cholelithiasis**

The Russian Gastrointestinal Association created a set of CPGs on cholelithiasis in 2015 [16]. It is 37 pages long (2-line spacing), which includes the title page and 22 references. From those references, 5 are in Russian and 17 are in English. The in-text citations are only present in the section on the history of research on cholelithiasis and on one figure. This is the first CPG we studied that includes multiple descriptive and colourful figures. The text size and spacing between paragraphs is inconsistent, so it is hard to tell which sections are the main ones. The CPG begins with a title page, which states that the CPG was developed by the gastroenterology specialists of the Russian Ministry of Healthcare together with the Russian Gastrointestinal Association. The next page begins with the list of authors and their academic level (academician, professor, or associate professor). There are 4 authors. Next, there is a list of abbreviations used in text and their meaning.

The CPG begins with a section titled *Methodology*. It consists of methods used for collecting and selecting data, methods used for assessing the quality and strength of evidence, methods used for formulating the guidelines, and method of validation of guidelines (this is the only CPG that includes external expert assessment). Next, there is a translated into Russian table with the levels of evidence according to the Oxford Centre for Evidence-based Medicine that has a range of evidence between 1a and 4. There is also a rating table for assessing the strength of recommendations which ranges from A to D and mentions the 1++ to 2+ scale in the description. The text of the CPG does not refer back to either of those tables or mention any one of the possible values from the tables.

The first half of the CPG has information on epidemiology of cholelithiasis in Russia and other countries, history of research of this pathology, and conditions needed for gallstones to appear (which includes multiple descriptive tables and figures). Next, there is a section titled Clinical presentation, which describes different options of how cholelithiasis can progress and some of the most common treatments. Next is the differential diagnosis with multiple descriptive tables as well as general diagnosis with many diagnostic options. Next is the longest section on *treatment strategy*, which includes: *lifestyle and nutrition*; *surgical treatment*; *the concept of post-cholecystectomy syndrome*; *minimally invasive interventions*; and *conservative treatment*. Last is the *prognosis* section with a descriptive figure.

**Acute pancreatitis**

The Russian Society of Surgeons, the Association of Hepatopancreatic Surgeons of CIS Countries, and the Russian Society of Emergency Medicine created a collective set of CPGs for treating acute pancreatitis in 2014 [19]. It is 32 pages long (1.15-line spacing), which includes the title page and 18 references. From those references, 14 are in Russian and 4 are in English (but the reference title is translated into Russian). There are no in-text citations or any mention of the references. The CPG begins with a title page, which lists the 3 organizations that participated in the creation of the guidelines, but the authors are not listed anywhere in the document. The spacing between paragraphs and the font size of titles is irregular, so it is difficult to distinguish sections and their subsections from one another.

The body of the document begins with an *introduction* section, which states what CPGs are, how they are used, and why they are important. Next is the *definition and classification* of the pathology. The classification includes 3 types of acute pancreatitis based on the severity. Next are etiological forms of acute pancreatitis, pathogenesis, definition of the onset of acute pancreatitis, phases of acute pancreatitis, terminology, and primary and emergency medical care for acute pancreatitis. After that the document is divided into 2 main sections: *early* and *late* *phases* of the disease.

First is the section called *early phase of acute pancreatitis*. Throughout this section there are red bold and italicized phrases stating “strength of recommendation” and a value between A and D. There is no reference to where the rating comes from, but a table with the grading system for the strength of recommendations is included at the very end of the CPG. There is no reference in regard to where the table comes from. In the beginning of *early phase of acute pancreatitis* there are 6 parts that deal with different levels of the pathology and diagnosis methods. They are: *primary diagnostic protocol and treatment strategies; protocol of treatment of acute pancreatitis of low severity; protocol of intensive treatment of acute pancreatitis of medium severity; protocol of intensive treatment of acute pancreatitis of high severity; protocol of laparoscopic surgery;* and *computer tomography*. The next subsection is called *protocols of diagnosis and treatment of acute pancreatitis in the early phase of the disease*. It consists of 2 parts: *protocol of diagnosis and monitoring of peripancreatic infiltrate* and *protocol of treatment strategy to peripancreatic infiltrate*.

Next is the section called *late phase of acute pancreatitis*. It consists of 2 parts: *protocols of diagnosis and treatment of acute pancreatitis in the aseptic sequestration phase* (which includes *protocol of diagnosis and monitoring of pancreatic pseudocysts* and *protocol of treatment of pseudocysts of the pancreas*) and *protocols of diagnosis and treatment of acute pancreatitis in the septic sequestration phase* (which includes *protocol of diagnosis of purulent complications of acute pancreatitis* and *protocol of treatment of purulent complications of acute pancreatitis*).

The last section in the CPG is called Methodology, which includes the previously mentioned tables on levels of evidence (1++ to 4) and strength of recommendation (A through D). Under the tables, it is stated that the strength of recommendations and the level of evidence is included when the recommendations are given in the body of the text.

**Chronic pancreatitis**

The Russian Society of Surgeons and the Association of Hepatopancreatic Surgeons of CIS Countries created a collective set of CPGs for treating chronic pancreatitis in 2014 [21]. It is 49 pages long (1.5-line spacing), which includes the title page and 60 references. From those references, 14 are in Russian and 46 are in English. The in-text citations are consistently used throughout the text. The CPG begins with a title page, which is followed by a list of authors, which includes a project coordinator (1 person), work coordinators (2), expert committee (19), workgroup for guideline creation (11) for a total of 33 authors. Their affiliated cities and academic level (academician, DSc or PhD) are also listed. After that there is a table of contents. Not all items from the text are mentioned in the table of contents. Next, there is a list of abbreviations used throughout the CPG and a section on the goals of the CPG and its intended use. Next, there is a table with the strength of recommendations (classes I-IV) and a table with the levels of evidence (A-D). It is stated that these tables are internationally recognized but there is no reference as to where they are taken from. References to those tables, levels of evidence, and strength of recommendations are made consistently throughout the text. After that there is an explanation of the search strategy for evidence and how the CPG will be updated.

The rest of the CPG is divided in 7 sections that are conveniently mentioned in the table of contents. At the end of most sections and their subsections, there is a bolded statement (usually between 1-4 paragraphs) that is titled “recommendations” (рекомендации) with explicit guidelines and a level of evidence (A-D). The 7 main sections are: *definition*; *epidemiology*; *etiology and pathogenesis*; *classification of chronic pancreatitis* (which includes a descriptive table with ICB-10 codes that are related to pancreatic conditions and a table with the Marseilles-Rome international classification); *diagnosis* (which includes the natural course of chronic pancreatitis, diagnosis of chronic pancreatitis and its complications, *ultrasound*, *endoscopic ultrasound*, *computer tomography*, *magnetic resonance imaging*, and *esophagogastroduodenoscopy*); *endoscopic and surgical treatment of patients with chronic pancreatitis* (which includes *preoperative preparation*, *drainage surgeries*, *resective interventions*, and *endovascular interventions for false aneurisms and combined radical treatment*); and *postoperative complications*. Last is the conclusion section, where the CPG is summarized.
