# Supplementary material for: Assessing methodological quality of Russian clinical practice guidelines and introducing AGREE II instrument in Russia
Source: PLoS One. 2018 Sep 11;13(9):e0203328. doi: 10.1371/journal.pone.0203328 (PMC6133363; doi:10.1371/journal.pone.0203328)
Supplement: S2 Text — (PDF) [file pone.0203328.s002.pdf]

Ministry of Education and Science of Russian Federation
Federal State Autonomous Educational Institution of Higher Learning

Kazan Federal UNIVERSITY | INSTITUTE of Fundamental Medicine and Biology

420012, Kazan, K.Marx, 18
tel.: +7 (843) 236-76-40
fax.: +7 (843) 233-78-14
e-mail: medbiol@kpfu.ru

To whom it may concern

This is to certify that Prof. Liliya E. Ziganshina receives partial salary from the Russian Government Program of Competitive Growth of Kazan Federal University (https://kpfu.ru/eng/strategy-5-top-100/roadmap-in-details) for development of Cochrane Russia.

Due to the deepening of financial crisis, the grant awarded for the year 2018, of 2 019 020,00 Rubles to Research & Education Centre Cochrane Russia provides insufficient funding for sustaining Cochrane Russia staff members at basic part-time salaries.

Unfortunately we are unable at this time to provide support for publication fee assistance.

Albert A. Rizvanov, Ph.D., Dr.Sci.

Director of Program of Competitive Growth of Institute of Fundamental Medicine and Biology,
Vice-Director of Strategic Academic Unit "Translational 7P Medicine",
Corresponding member of the Tatarstan Academy of Sciences,
Chief researcher, professor of the Department of Genetics,
Institute of Fundamental Medicine and Biology,
Head of the Department of Exploratory Research, Scientific and Educational Center of Pharmaceutics, Kazan (Volga Region) Federal University, Kazan, Russia
rizvanov@gmail.com, albert.rizvanov@kpfu.ru
