# Supplementary material for: Assessing methodological quality of Russian clinical practice guidelines and introducing AGREE II instrument in Russia
Source: PLoS One. 2018 Sep 11;13(9):e0203328. doi: 10.1371/journal.pone.0203328 (PMC6133363; doi:10.1371/journal.pone.0203328)
Supplement: S3 Text — (DOCX) [file pone.0203328.s003.docx]

Оценочный инструмент анализа руководств (клинических рекомендаций)

**A**PPRAISAL OF **G**UIDELINES

FOR **RE**SEARCH & **E**VALUATION **II**

**Инструмент**

Консорциум The AGREE Next Steps

Май 2009

**ОБНОВЛЕНИЕ: Декабрь 2017**

АВТОРСКИЕ ПРАВА И ВОСПРОИЗВЕДЕНИЕ

Этот документ является продуктом международного сотрудничества.

Он может быть воспроизведен и использован в образовательных целях,

в программах обеспечения качества и критической оценки

клинических рекомендаций. Он не может быть использован для коммерческих

или маркетинговых целей. Должны использоваться только

одобренные не-англоязычные версии инструмента AGREE II,

когда таковые имеются. Предложения о помощи в переводе на другие

языки приветствуются, если они соответствуют

протоколу, установленному "The AGREE Research Trust".

ЗАЯВЛЕНИЕ ОБ ОТВЕТСТВЕННОСТИ

Инструмент AGREE II - это универсальный инструмент, предназначенный, прежде всего,

для того чтобы помочь разработчикам рекомендаций и пользователям оценивать

методологическое качество рекомендаций.

Авторы не берут на себя ответственность за неправильное использование инструмента AGREE II.

© The AGREE Research Trust, May 2009.

© The AGREE Research Trust, September 2013.

© AGREE Enterprise December 2017.

ПРЕДЛАГАЕМАЯ ССЫЛКА ДЛЯ ЦИТИРОВАНИЯ AGREE II

Brouwers MC, Kho ME, Browman GP, Burgers JS, Cluzeau F, Feder G, Fervers B, Graham ID, Grimshaw J, Hanna

SE, Littlejohns P, Makarski J, Zitzelsberger L, for the AGREE Next Steps Consortium. AGREE II: Advancing guideline

development, reporting and evaluation in healthcare. CMAJ 2010;182:E839-842.

ПРЕДЛАГАЕМАЯ ССЫЛКА ДЛЯ ВЕРСИИ AGREE II PDF

Next Steps Consortium (2017). *The AGREE II Instrument* [Electronic version]. Retrieved <Month, Day,

Year>, from <http://www.agreetrust.org>.

ФИНАНСИРОВАНИЕ

Разработка инструмента AGREE II финансировалась

грантом Канадских институтов исследований в области здравоохранения (FRN77822)

ЗА ДОПОЛНИТЕЛЬНОЙ ИНФОРМАЦИЕЙ О AGREE II, ПОЖАЛУЙСТА ОБРАЩАЙТЕСЬ

AGREE Project Office, agree@mcmaster.ca

AGREE Website, www.agreetrust.org

Перевод на русский язык выполнен командой Кокрейн Россия (Cochrane Russia)

в Казанском федеральном университете:

Рашида М. Хамидулина (перевод и форматирование), Лидия В. Кисарь (перевод),

Чарльз Х.В. Хойл (Charles H.V. Hoyle) – обратный перевод на английский язык, сверка текстов, редактирование, Лилия Е. Зиганшина (редактирование и координация работы по переводу).

| **CОСТАВ КОНСОРЦИУМА AGREE NEXT STEPS** |
| --- |

Dr. Melissa C. Brouwers

Principal Investigator, AGREE Next Steps Consortium

McMaster University, Hamilton, Ontario, Canada

Участники Консорциума:

Dr. G.P. Browman, Victoria, British Columbia, Canada

Dr. J.S. Burgers, Dutch College of General Practitioners, Utrecht, The Netherlands

Dr. F. Cluzeau, Global Health and Development Group, Imperial College London, UK

Dr. D. Davis, Association of American Medical Colleges, Washington DC, USA

Dr. G. Feder, University of Bristol, UK

Dr. B. Fervers, Cancer et Environement, Centre Léon Bérard, France

Dr. I. Graham, Ottawa Hospital Research Institute, Ottawa, Ontario, Canada

Dr. J. Grimshaw, Ottawa Hospital Research Institute, Ottawa, Ontario, Canada

Dr. S.E. Hanna, McMaster University, Hamilton, Ontario, Canada

Ms. M.E. Kho, McMaster University, Hamilton, Ontario Canada

Dr. P. Littlejohns, Kings College London, UK

Ms. J. Makarski, Hamilton, Ontario, Canada

Dr. L. Zitzelsberger, Quebec, Canada

| **ВЕРСИИ И ОБНОВЛЕНИЯ AGREE II**  Первоначальный публичный релиз и Дата публикации AGREE II: 2009/2010  Обновление AGREE II: Сентябрь 2013  **Обновление AGREE II: Декабрь 2017**  ***Что нового в обновлении от Декабря 2017?***  *Обновление от Августа 2017 года включает изменения в следующие разделы Введения: «AGREE веб-сайт: ресурсы и ссылки», «10 лет AGREE» и «Подсчет баллов AGREE II». Было добавлено руководство по использованию порогов оценки AGREE II, чтобы различать руководства более высокого качества от руководств низкого качества. Кроме того, незначительные редакционные изменения были внесены во Введение и Руководство пользователя. Содержание самого инструмента AGREE II не изменялось с 2009 года, и все версии AGREE II остаются в силе для использования.* |
| --- |

| **СОДЕРЖАНИЕ** |
| --- |

**I. ВВЕДЕНИЕ**

I. Обзор……………………………………………………………………………………..1

II. Применение AGREE II…………………………………………………………...........3

III. Сайт AGREE: Ресурсы и библиография…..…………………………………….….3

IV. 10 лет AGREE…………………………………………………………………………..4

**II. РУКОВОДСТВО ПОЛЬЗОВАТЕЛЯ: ИНСТРУКЦИИ ДЛЯ ИСПОЛЬЗОВАНИЯ AGREE II**……………………………………………………………………………………………6

I. Подготовка к использованию AGREE II……………………………………………7

II. Структура и содержание AGREE II…………………………………………………7

III. Шкала оценки и разделы руководства пользователя…………………………..8

IV. Подсчет баллов в AGREE II………………………………………………………….9

V. Общая оценка………………………………………………………………………...10

VI. Руководство по оценке каждого пункта

*а. Домен 1. Область применения и цель*………………………………..………11

*b. Домен 2. Участие заинтересованных сторон………………………...……*15

*c. Домен 3. Тщательность разработки………………………………………...*19

*d. Домен 4. Ясность изложения…………………………………………………..*.28

*e. Домен 5. Применение……………………………………………………….……*32

*f. Домен 6. Редакционная независимость………………………………………*37

*g. Общая оценка руководства*…………………………………………………….40

**III. Инструмент AGREE II**…………………………………………………………..................42

1. Домен 1. Область применения и цель……………………………………………43
2. Домен 2. Участие заинтересованных сторон……………………………………44
3. Домен 3. Тщательность разработки………………………………………………45
4. Домен 4. Ясность изложения………………………………………………….……48
5. Домен 5. Применимость….....…………………………………………………..…49
6. Домен 6. Редакционная независимость……………………………………….…51
7. Общая оценка руководства…………………………………………………………52

**I. ВВЕДЕНИЕ**

| **I. ОБЗОР** |
| --- |

**i) Цель инструмента AGREE II**

Руководства по клинической практике (руководства или клинические рекомендации) — это систематически разработанные положения для помощи в принятии решений практикующему врачу и пациенту о надлежащей медицинской помощи в конкретных клинических ситуациях (1). Дополнительно, рекомендации могут играть важную роль в формировании политики здравоохранения (2,3) и эволюционировали, охватив в целом все темы континуума здравоохранения (например, укрепление здоровья, скрининг, диагностика).

Потенциальная польза от руководств зависит от качества самого руководства. Для успешной реализации итоговых рекомендаций (4-6) важны соответствующие методологии и четкие стратегии в процессе разработки руководств. Качество клинических руководств может быть чрезвычайно вариабельным, а некоторые часто не соответствуют базовым стандартам (7-9).

Оценочный инструмент анализа клинических рекомендаций AGREE II (**A**ppraisal of **G**uidelines for **RE**search & **E**valuation (AGREE) Instrument) (10) был разработан для решения проблемы различного качества руководства. С этой целью, инструмент AGREE – средство оценки методологической тщательности и прозрачности, использованных при разработке руководства. Оригинальный инструмент AGREE был усовершенствован до инструмента AGREE II, также было разработано Руководство пользователя (11-13).

Цель AGREE II заключается в том, чтобы обеспечить основу для следующих действий:

1. Оценить качество руководств;
2. Обеспечить методологическую стратегию разработки клинических рекомендаций (руководств);
3. Показать, какая информация и как информация должна быть представлена в руководствах.

AGREE II заменяет первоначальный инструмент и является предпочтительным, и может использоваться как часть общего мандата качества, направленного на улучшение здравоохранения.

- 1. **История проекта AGREE**

Первоначальный инструмент AGREE был опубликован в 2003 году группой международных разработчиков и исследователей, Сотрудничество AGREE (10). Целью Сотрудничества было разработать инструмент для оценки качества клинических рекомендаций. Сотрудничество AGREE определило качество руководств *как уверенность в том, что все потенциальные смещения (предвзятость) в разработке руководства были адекватно рассмотрены, и что рекомендации валидны, как внутренне, так и внешне, и выполнимы в практике* (10). Оценка включает суждения о методах, использованных для разработки руководств, компонентов итоговых рекомендаций, и факторов, которые связаны с их внедрением. Результатом усилий Сотрудничества стал первоначальный инструмент AGREE, инструмент из 23 пунктов, составляющих 6 доменов (разделов) качества. Инструмент AGREE был переведен на многие языки, упоминается в более чем 600 публикациях и одобрен несколькими организациями здравоохранения. Более подробная информация об инструменте и соответствующих публикациях доступна на веб-сайте AGREE (<http://www.agreetrust.org/>).

Как и в случае любого нового инструмента оценки, было признано, что необходима его продолжающаяся разработка и развитие для укрепления измеряющих свойств инструмента и для обеспечения удобства его использования и практической жизнеспособности среди предполагаемых пользователей. Это побудило нескольких членов первоначальной команды организовать Консорциум дальнейших шагов (Консорциум AGREE Next Steps). Цели Консорциума заключались в дальнейшем улучшении измеряющих свойств инструмента, включая его надежность и достоверность; уточнении пунктов для более полного удовлетворения потребностей предполагаемых пользователей; и улучшении вспомогательной документации (т. е. оригинального учебного пособия и руководства пользователя) с тем, чтобы дать пользователям возможность использовать инструмент с уверенностью.

Результатом этих усилий является AGREE II, который состоит из обновленного Руководства пользователя и Инструмента, состоящего из 23 пунктов, составляющих те же шесть доменов, описанных здесь. Руководство пользователя представляет собой существенную модификацию первоначального учебного пособия и руководства пользователя и содержит подробную информацию по каждому из 23 пунктов. В таблице 1 сравниваются пункты первоначальной версии с пунктами версии AGREE II.

Таблица 1. Сравнение пунктов первоначальной версии AGREE и AGREE II.

| **Пункты первоначальной версии AGREE** | | | **Пункты AGREE II** |
| --- | --- | --- | --- |
| **Домен 1. Область применения и цель** | | | |
| 1. | Общая (-ие) цель (-и) клинического руководства подробно описана (-ы): | | Без изменений |
| 2. | Клинический (-е) вопрос (-ы), рассматриваемый (-е) в руководстве, подробно описан (-ы): | | Вопросы здоровья и здравоохранения, рассматриваемые в руководстве, подробно описаны. |
| 3. | Пациенты, к которым руководство будет применяться, описаны точно. | | Популяция (пациенты, общество), к которым руководство будет применяться, описаны точно. |
| **Домен 2. Участие заинтересованных сторон** | | | |
| 4. | Группа разработчиков руководства включает специалистов из всех соответствующих профессиональных групп: | | Без изменений |
| 5. | Были изучены взгляды и предпочтения пациентов. | | Были изучены взгляды и предпочтения целевой популяции (пациентов, общественности и т. д.) |
| 6. | Четко определена целевая аудитория (пользователи) руководства. | | Без изменений |
| 7. | Руководство было опробовано среди конечных пользователей | | Пункт удален. Включено в руководство пользователя в описании пункта 19. |
| **Домен 3. Тщательность разработки** | | | |
| 8. | Использованы систематические методы поиска доказательств | | Без изменений, перенумерован в пункт 7. |
| 9. | Критерии отбора доказательств четко описаны: | | Без изменений, перенумерован в пункт 8. |
|  |  | | **НОВЫЙ** пункт 9. Сила и ограничения совокупности доказательств четко описаны. |
| 10. | Четко описаны методы составления и формулирования рекомендаций. | | Без изменений |
| 11. | Польза для здоровья, побочные эффекты и риски предлагаемых подходов были рассмотрены при составлении и формулировании рекомендаций. | | Без изменений |
| 12. | Имеется недвусмысленная связь между рекомендациями и подкрепляющими их доказательствами | | Без изменений |
| 13. | Руководство было рецензировано внешними экспертами до публикации. | | Без изменений |
| **Пункты первоначальной версии AGREE** | | **Пункты AGREE II** | |
| 14. | Предусмотрена процедура обновления руководства | Без изменений | |
| **Домен 4. Ясность изложения** | | | |
| 15. | Рекомендации конкретны и недвусмысленны. | Без изменений | |
| 16. | Четко описаны различные варианты ведения соответствующего состояния. | Четко описаны различные варианты ведения соответствующего состояния или проблемы здоровья. | |
| 17. | Легко выделить/определить ключевые рекомендации. | Без изменений | |
| **Домен 5. Применение/Внедрение** | | | |
| 18. | Руководство содержит инструменты для внедрения | Руководство предоставляет советы и / или инструменты о том, как рекомендации могут быть реализованы на практике. И Изменение в Домене (с Ясности представления) и перенумерован в пункт 19. | |
| 19. | Потенциальные организационные ограничения при применении рекомендации были обсуждены | В руководстве описаны способствующие факторы (фасилитаторы) и барьеры для его применения  **И** изменение порядкового номера - перенумеровано в пункт 18 | |
| 20. | Рассмотрены возможные финансовые последствия применения рекомендаций. | Рассмотрены возможные ресурсные / финансовые последствия применения рекомендаций. | |
| 21. | В руководстве представлены ключевые критерии оценки для целей мониторинга и/или аудита | В руководстве представлены критерии для мониторинга и / или аудита. | |
| **Домен 6. Редакционная независимость** | | | |
| 22. | Руководство является редакционно независимым от финансирующей организации | Взгляды финансирующей организации не повлияли на содержание руководства. | |
| 23. | Конфликт интересов членов группы разработчиков были документированы. | Конкурирующие интересы членов группы по разработке руководства были документированы и учтены. | |

| **II. ПРИМЕНЕНИЕ AGREE II** |
| --- |

1. **Какие клинические руководства могут быть оценены инструментом AGREE II?**

Как и в случае с первоначальным инструментом, AGREE II предназначен для оценки клинических руководств, разработанных местными, региональными, национальными или международными группами, или аффилированными правительственными организациями. К ним относятся оригинальные версии и обновления существующих руководств.

Инструмент AGREE II универсальный и может быть применен к руководствам в любой области здравоохранения или в клинической области по заболеваниям, обращенным к любой ступени континуума здравоохранения, включая пропаганду здоровья, общественное здоровье и здравоохранение, скрининг, диагностику, лечение или вмешательства. Инструмент подходит для руководств, представленным в бумажном и электронном виде. AGREE II не предназначен для оценки качества руководств, направленных на решение организационных вопросов здравоохранения. Его роль в анализе оценок медицинских технологий формально не была проведена.

**ii) Кто может использовать AGREE II?**

AGREE II предназначен для использования следующими группами заинтересованных:

- **работникам здравоохранения**, которые хотят провести собственную оценку руководства, прежде чем применять рекомендации руководства в своей практике;
- **разработчиками руководств**, чтобы придерживаться четкой и строгой методологии разработки, чтобы провести внутреннюю оценку для обеспечения правильности их рекомендаций; или для оценки рекомендаций других групп для потенциальной адаптации к собственной практике;
- **определяющими политику здравоохранения**, чтобы помочь им решить, какие руководства могут быть рекомендованы для использования на практике или для принятия обоснованных политических решений;
- **преподавателями**, чтобы помочь повысить навыки критической оценки среди специалистов в области здравоохранения и обучить основным компетенциям в разработке руководства и отчетности.

| **III. Сайт AGREE: РЕСУРСЫ И ССЫЛКИ** |
| --- |

Веб-сайт Инициативы AGREE [www.agreetrust.org](http://www.agreetrust.org), содержит множество инструментов для помощи пользователям использовать AGREE II.

1. **Публикации Исследований AGREE**

- Доступ к публикациям, связанным с AGREE II и другими AGREE инструментами.
- ***Основные публикации*:** страница AGREE II предоставляет доступ к публикациям, связанным с разработкой и тестированием AGREE II.

1. **Инструменты обучения AGREE II**

- Для обучения новых пользователей AGREE II доступны два онлайн-инструмента:
- Обзорное учебное пособие AGREE II,
- Практическое упражнение AGREE II.

1. **Переводы AGREE II**

- Благодаря участникам международного сообщества клинических руководств AGREE II был переведен на разные языки.
- Копии переводов доступны широкой общественности на этом сайте.
- Если вы хотите сделать новый перевод, пожалуйста, свяжитесь с офисом проекта AGREE по электронному адресу [agree@mcmaster.ca](mailto:agree@mcmaster.ca).

1. **Мой AGREE PLUS**

- Онлайн платформа My AGREE PLUS в свободном публичном доступе для заполнения и отслеживания опросников AGREE II.
- Платформа может быть использована для:
- Индивидуального заполнения опросника AGREE II,
- Участия в группе по оценке AGREE II,
- Координации группы по оценке AGREE II.
- **Перейти по ссылке MY AGREE PLUS** [www.agreetrust.org](http://www.agreetrust.org) для регистрации и использования платформы.

1. **Другие инструменты AGREE**

- Доступ к другим инструментам AGREE для поддержки разработки, отчетности и оценки клинических рекомендаций и руководств по системам здравоохранения:
- **Контрольный список (чек-лист) отчетности AGREE**: чек-лист на основе AGREE II для ведения отчетности о клинических руководствах (14).
- **AGREE GRS**: инструмент из 4 пунктов для оценки качества рекомендаций, когда нехватка времени или ресурсов делает невозможным использование полной версии AGREE II.
- **Рекомендации AGREE Excellence (AGREE-REX):** инструмент для оценки качества и для направления разработки и отчетности рекомендаций клинического руководства.
- **AGREE Health Systems (AGREE-HS):** инструмент для оценки качества и для направления разработки и отчетности руководящих документов систем здравоохранения.
- **CheckUp:** контрольный список для составления отчетов по обновлённым клиническим руководствам (15).

| **IV. 10 лет AGREE** |
| --- |

В 2013 году Инициатива AGREE отметила свой 10-летний юбилей, когда инструмент AGREE был впервые опубликован и стал доступен для использования. Чтобы отметить этот юбилей, была опубликована статья, подводившая итоги научного путешествия AGREE, отметив, что многие достижения на пути (16).

**ССЫЛКИ**

1. Woolf SH, Grol R, Hutchinson A, Eccles M, Grimshaw J. Clinical guidelines: potential

benefits, limitations, and harms of clinical guidelines. *BMJ.* 1999;318(7182):527-530.

2. Committee to Advise the Public Health Service on Clinical Practice Guidelines IoM. *Clinical*

*practice guidelines: directions for a new program.* Washington: National Academy Press;

1990.

3. Browman GP, Snider A, Ellis P. Negotiating for change. The healthcare manager as catalyst

for evidence-based practice: changing the healthcare environment and sharing experience.

*Healthc Pap.* 2003;3(3):10-22.

4. Grol R. Success and failures in the implementation of evidence-based guidelines for clinical

practice. *Med Care*. 2001;39(8 Suppl 2):1146-54.

5. Davis DA, Taylor-Vaisey A. Translating guidelines into practice: a systematic review of

theoretic concepts, practice experience and research evidence in the adoption of clinical

practice guidelines. *CMAJ*. 1997;157(4):408-16.

6. Grimshaw J,.Russell I. Effect of clinical guidelines on medical practice: a systematic review

of rigorous evaluations. *Lancet*. 1993; 342:1317-22.

7. Shaneyfelt TM, Mayo-Smith MF Rothwangl J. Are guidelines following guidelines? The

methodological quality of clinical practice guidelines in the peer-reviewed medical literature*.*

*JAMA* 1999:281(20):1900-5.

8. Grilli R, Magrini N, Penna A, Mura G, Liberati A. Practice guidelines developed by specialty

societies: the need for critical appraisal. *Lancet.* 2000; 355:103-6.

9. Burgers JS, Fervers B, Haugh M, Brouwers M, Browman G, Phillip T, Cluzeau FA.

International assessment of the quality of clinical practice guidelines in oncology using the

Appraisal of Guidelines and Research and Evaluation Instrument. *J Clin Oncol*.

2004; 22:2000-7.

10. AGREE Collaboration. Development and validation of an international appraisal instrument

for assessing the quality of clinical practice guidelines: the AGREE project. *Qual Saf Health*

*Care*. 2003;12(1):18-23.

11. Brouwers MC, Kho ME, Browman GP, Burgers JS, Cluzeau F, Feder G, Fervers B, Graham

ID, Grimshaw J, Hanna S, Littlejohns P, Makarski J, Zitzelsberger L, for the AGREE Next

Steps Consortium. AGREE II: advancing guideline development, reporting and evaluation in

health care. *CMAJ.* 2010;182(18):E839-42.

12. Brouwers MC, Kho ME, Browman GP, Burgers JS, Cluzeau F, Feder G, Fervers B, Graham

ID, Hanna SE, Makarski J, for the AGREE Next Steps Consortium. Development of the

AGREE II, part 1: performance, usefulness and areas for improvement. CMAJ

2010;182(10):1045-52.

13. Brouwers MC, Kho ME, Browman GP, Burgers J, Cluzeau F, Feder G, Fervers B, Graham

ID, Hanna SE, Makarski J, for the AGREE Next Steps Consortium. Development of the

AGREE II, part 2: assessment of validity of items and tools to support application. CMAJ

2010;182(10): E472-8.

14. Brouwers MC, Kerkvliet K, Spithoff K, on behalf of the AGREE Next Steps Consortium. The

AGREE Reporting Checklist: A tool to improve reporting of clinical practice guidelines. BMJ

2016;352: i1152.

15. Vernooij RWM, Alonso-Coello P, Brouwers M, Martinez Garcia L, CheckUp Panel. Reporting

items for updated clinical guidelines: Checklist for the Reporting of Updated Guidelines

(CheckUp). PLOS Medicine 2017;14(1): e1002207.

16. Makarski J, Brouwers MC. The AGREE Enterprise: a decade of advancing clinical practice

guidelines. Implement Sci. 2014; 9:103.

| **AGREE II:**  **РУКОВОДСТВО ПОЛЬЗОВАТЕЛЯ** |
| --- |

**II. РУКОВОДСТВО ПОЛЬЗОВАТЕЛЯ: ИНСТРУКЦИИ ДЛЯ ИСПОЛЬЗОВАНИЯ AGREE II.**

Настоящее Руководство пользователя специально разработано для того, чтобы ориентировать оценивающих в использовании инструмента. Мы рекомендуем прочитать следующие инструкции перед использованием инструмента.

| **I. Подготовка к использованию AGREE II** |
| --- |

**i) Сопроводительные документы**

Перед применением AGREE II пользователи должны вначале тщательно полностью ознакомиться с текстом руководства. В дополнение к основному документу пользователи должны попытаться идентифицировать всю информацию о процессе разработки руководства до оценки. Эта информация может содержаться в том же документе, что и рекомендации руководства, или может быть суммирована в отдельном техническом отчете, методическом руководстве или директивном заявлении разработчика. Эти подтверждающие документы могут быть опубликованы или могут быть доступны публично на веб-сайтах. Хотя это ответственность авторов рекомендаций, информировать пользователей о наличии и местонахождении соответствующих дополнительных технических и вспомогательных документов, пользователям AGREE II следует приложить все усилия, чтобы найти и включить их как часть материалов, подходящих для оценки.

**ii) Число (оценивающих) экспертов**

Мы рекомендуем, чтобы каждое руководство оценивалось, по меньшей мере, двумя экспертами, а предпочтительно 4, как так это повысит надежность оценки.

| **II. Структура и Содержание AGREE II** |
| --- |

AGREE II состоит из 23 пунктов, объединённых в 6 доменов и завершающих 2 общих пунктов оценки (Общая оценка). Каждый домен фиксирует отдельную сферу качества руководства.

*ДОМЕН 1. ОБЛАСТЬ ПРИМЕНЕНИЯ И ЦЕЛЬ* касается общей цели руководства, конкретного вопроса здоровья / здравоохранения и целевой популяции (пункты 1-3).

*ДОМЕН 2. УЧАСТИЕ ЗАИНТЕРЕСОВАННЫХ СТОРОН* фокусирует внимание на том, в какой степени руководство было разработано соответствующими заинтересованными сторонами и представляет взгляды предполагаемых пользователей (пункты 4-6).

*ДОМЕН 3. ТЩАТЕЛЬНОСТЬ РАЗРАБОТКИ* относится к процессу, использованному для сбора и синтеза доказательства, методам формулирования рекомендаций и их обновления (пункты 7-14).

*ДОМЕН 4. ЯСНОСТЬ ИЗЛОЖЕНИЯ* касается языка, структуры и формата руководства (пункты 15-17).

*ДОМЕН 5. ПРИМЕНЕНИЕ / ВНЕДРЕНИЕ* относится к вероятным барьерам и фасилитаторам для реализации, стратегиям для улучшения восприятия и ресурсным последствиям применения руководства (пункты 18-21).

*ДОМЕН 6. РЕДАКЦИОННАЯ НЕЗАВИСИМОСТЬ* рассматривает формулирование рекомендаций, чтобы они не были не долженствующим образом смещены (предвзяты) из-за конкурирующих интересов (пункты 22-23).

*ОБЩАЯ ОЦЕНКА* включает в себя общий рейтинг качества руководства, и будет ли руководство рекомендовано для использования в практике.

| **III. Шкала оценки и разделы Руководства Пользователя** |
| --- |

Каждый из пунктов AGREE II и два общих пункта оцениваются по 7-балльной шкале (1 – совершенно не согласен и 7 – совершенно согласен). В Руководстве пользователя приведены рекомендации о том, как оценивать каждый пункт с использованием шкалы оценки, а также включает в себя 3 дополнительных раздела для дальнейшего облегчения оценки пользователем. Разделы включают Описание, Где искать, Как оценивать.

**i) Шкала оценки**

Все пункты AGREE II оцениваются по 7-бальной шкале:

| **1** Совершенно не согласен (-на) | **2** | **3** | **4** | **5** | **6** | **7** Совершенно согласен (-на) |
| --- | --- | --- | --- | --- | --- | --- |

Оценка 1 (*Совершенно не согласен*). Оценку 1 следует дать, когда нет информации, имеющей отношение к пункту AGREE II, плохо представлена концепция, или авторы прямо заявляют, что критерии не были соблюдены.

Оценка 7 (*Совершено согласен*). Оценку 7 следует дать, если качество отчетности является исключительным и соблюдены все критерии и соображения, изложенные в Руководстве пользователя.

Оценки между 2 и 6. Оценка между 2 и 6 дается, когда описываемый пункт AGREE II не соответствует полностью всем критериям или соображениям. Оценка дается в зависимости от полноты и качества представления информации. Оценки увеличиваются по мере соответствия большему числу критериев и соображений. Раздел «Как оценивать» для каждого пункта включает сведения о критериях оценки и конкретных соображениях.

**ii) Описание**

Этот раздел определяет концепцию, лежащую в основе этого пункта в широком смысле, и дает примеры.

**iii) Где искать**

Этот раздел направляет пользователя, где обычно можно найти информацию в руководстве. В этот раздел включены общие термины, используемые для обозначения разделов или глав руководства. *Это только предложения*. Ответственность за оценку всего руководства и сопроводительных материалов несет оценивающий эксперт, чтобы обеспечить справедливую оценку.

1. **Как оценивать**

В этом разделе приведены сведения о критериях оценки и особенностях каждого конкретного пункта.

- *Критерии* определяют точные элементы, которые отражают подходящее (рабочее) определение пункта. Чем больше критериев удовлетворяется, тем более высокую оценку руководство должно получить по этому пункту.
- *Дополнительные соображения* направлены на то, чтобы помочь информировать оценку. Как и в любой оценке, необходимы суждения оценивающих экспертов). Чем больше соображений (мнений) было учтено в руководстве, тем более высокую оценку руководство должно получить по этому пункту.

Важно отметить, что оценка руководств требуют определенного уровня суждений. Критерии и соображения предназначены для помощи, но не для замены этих суждений. Таким образом, ни один из пунктов AGREE II не дает ясных ожиданий по каждому из 7 баллов шкалы.

1. **Другие соображения при использовании AGREE II**

Иногда, некоторые пункты AGREE II могут быть не применимы к конкретному рассматриваемому руководству. Например, узкопрофильные руководства могут не предоставить весь спектр вариантов ведения состояния (см. пункт 16). AGREE II не включает в шкалу оценки ответ «Не применимо». Существуют различные стратегии управления этой ситуацией, в том числе, когда эксперты пропускают этот пункт в процессе оценки или оценивают пункт как 1 (отсутствие информации) и предоставляют комментарий по поводу оценки. *Независимо от выбранной стратегии, решения должны приниматься заранее, описываться подробно, и если пункты пропускаются, должны быть внесены соответствующие изменения в расчет оценок раздела. В принципе, исключение предметов из процесса оценки не рекомендуется.*

| **IV. Подсчет баллов в AGREE II** |
| --- |

Оценка качества в баллах рассчитывается для каждого из шести доменов AGREE II. Шесть оценок разделов являются независимыми и не должны быть объединены в единый показатель качества.

**i) Расчет оценки домена**

Оценки в баллах каждого домена рассчитываются путем суммирования всех баллов за отдельные пункты домена и вычисления процента от максимально возможного балла за весь домен.

**Например:**

Если 4 эксперта дают следующие баллы для Домена 1 (Область применения и цель):

|  | Пункт 1 | Пункт 2 | Пункт 3 | **Общее** |
| --- | --- | --- | --- | --- |
| Эксперт 1 | 5 | 6 | 6 | **17** |
| Эксперт 2 | 6 | 6 | 7 | **19** |
| Эксперт 3 | 2 | 4 | 3 | **9** |
| Эксперт 4 | 3 | 3 | 2 | **8** |
| **Общее** | **16** | **19** | **18** | **53** |

Максимально возможная оценка = 7 (совершенно согласен) * 3 (пункта) * 4 (эксперта) = 84

Минимально возможная оценка = 1 (совершенно не согласен) * 3 (пункта) * 4 (эксперта) = 12

Оценка домена в баллах будет следующая:

| Полученный бал - Минимально возможный бал | |
| --- | --- |
| Максимально возможный бал - Минимально возможный бал | |

| 53-12 |  | 41 |  |  |  |  |  | |
| --- | --- | --- | --- | --- | --- | --- | --- | --- |
| 84-12 | х 100 = | 72 | х 100 = 0,5694 х 100 = 57% | | | | |  |

*Если пункт не включен в оценку (пропущен), необходимы соответствующие изменения в подсчете максимально и минимально возможных оценок.*

ii) Интерпретация Балльных Оценок Домена

Оценки доменов в баллах могут использоваться для определения сильных сторон и ограничений клинических руководств, для сравнения методологического качества руководств или выбора руководств высокого качества для адаптации, одобрения или реализации. В настоящее время нет эмпирических данных для связи определенной оценки качества с конкретными результатами имплементации (например, скорость принятия, распространение принятия) или конкретные клинические исходы; это делает выбор пороговых оценок качества руководств для различения высокого, среднего и низкого качества сложным. При отсутствии этих данных, мы приводим примеры подходов, которые могут использоваться для установки пороговых значений качества.

- Приоритизация одного Домена: посредством обсуждений или на основе решений руководства, один домен качества может быть приоритетным по сравнению с другими. Таким образом, пороги могут быть созданы на основе оценок для приоритетного домена (например, руководства высокого качества – это те, что имеют оценку более 70% в Домене 3).
- Поэтапная оценка AGREE II: если пользователи рассматривают ценность одного домена как более высокую, чем других, они могут сначала оценить рекомендации, используя только этот домен. Только те руководства, которые соответствуют порогу качества для этого домена (например, больше 70%), затем оцениваются с использованием других пяти доменов AGREE II.
- Учет оценок всех домена: пользователи могут создать пороговые значения для всех шести доменов на основе консенсуса или решений руководства (например, руководствами высокого качества считать те, оценки всех доменов которых выше 70%). Альтернативно, пользователи могут установить разные пороги качества для каждого домена отдельно.
- Пороги для улучшения с течением времени: при анализе изменений в оценках руководств с течением времени, пользователи могут создавать пороговые значения для улучшения (например, минимум 10%-е улучшение в балльной оценке каждого домена руководства от конкретного разработчика за период времени в 5 лет).

Любые решения о том, как определять пороговые значения качества, должны приниматься группой всех соответствующих заинтересованных сторон до начала оценки AGREE II. Решения должны руководствоваться контекстом, в котором руководство будет использоваться, и оценкой важности разных доменов и пунктов в этом контексте.

| **V. Общая оценка** |
| --- |

По завершении 23 пунктов, пользователи AGREE II предоставят 2 общие оценки руководства. Общая оценка требует от пользователя вынесения суждения относительно качества с учетом критериев, рассмотренных в процессе оценки. Пользователю также задается вопрос, будет ли он рекомендовать руководство к использованию.

Следующие страницы включают, по доменам, направляющие советы по балльной оценке каждого из 23 пунктов AGREE II при анализе рекомендаций. Каждый пункт включает описание, предложения по нахождению информации по этому пункту, и советы, как оценивать.

| **ДОМЕН 1. ОБЛАСТЬ ПРИМЕНЕНИЯ И ЦЕЛЬ** |
| --- |

1. Общая (-ие) цель (-и) клинического руководства подробно описана (-ы).
2. Вопросы здоровья / здравоохранения, рассматриваемые в руководстве, подробно описаны.
3. Популяция (пациенты, общество и т.д.), к которой руководство будет применяться, описана точно.

**SCOPE AND PURP**

| **ОБЛАСТЬ ПРИМЕНЕНИЯ И ЦЕЛЬ**  **1. Общая (-ие) цель (-и) клинического руководства подробно описана (-ы):** |
| --- |

| **1** Совершенно не согласен (-на) | **2** | **3** | **4** | **5** | **6** | **7** Совершенно согласен (-на) |
| --- | --- | --- | --- | --- | --- | --- |

*Комментарии:*

ОПИСАНИЕ:

Целью этого пункта является оценка возможного влияния клинических рекомендаций на здоровье общества в целом и на отдельные популяции лиц или пациентов. Общие цели клинического руководства должны быть подробно описаны, а ожидаемая польза руководства должна быть специфичной для клинической проблемы или области здравоохранения. Примерами специфичных положений могут быть:

- Профилактика (долгосрочная) осложнений у больных сахарным диабетом
- Снижение риска сосудистых осложнений у больных, перенесших инфаркт миокарда
- Наиболее эффективная стратегия колоректального скрининга населения
- Предоставление рекомендаций по наиболее эффективному терапевтическому лечению и наблюдению пациентов с сахарным диабетом.

ГДЕ ИСКАТЬ:

Изучите вступительные параграфы / главы, чтобы найти описание области применения и цели руководства (клинических рекомендаций). В некоторых случаях обоснование или потребность в руководстве описывается в документе отдельно от руководства, например, в проекте руководства. Примеры разделов или глав в руководстве, где эта информация может быть найдена, включают: введение, область применения, целесообразность, обоснование, актуальность и цель.

КАК ОЦЕНИВАТЬ:

Содержимое раздела включает следующие КРИТЕРИИ:

- предназначение для здоровья (т.е. профилактика, скрининг, диагностика, лечение и т. д.)
- ожидаемая польза или исход
- на кого нацелено руководство (например, популяция пациентов, общество)

Дополнительные СООБРАЖЕНИЯ:

- Хорошо ли написан этот пункт? Являются ли описания ясными и краткими?
- Легко ли найти содержание этого пункта в руководстве?

| **ОБЛАСТЬ ПРИМЕНЕНИЯ И ЦЕЛЬ**  **2. Вопросы здоровья / здравоохранения, рассматриваемые в руководстве, подробно описаны:** |
| --- |

| **1** Совершенно не согласен (-на) | **2** | **3** | **4** | **5** | **6** | **7** Совершенно согласен (-на) |
| --- | --- | --- | --- | --- | --- | --- |

*Комментарии:*

ОПИСАНИЕ:

Должно быть подробное описание клинических вопросов, охватываемых руководством, особенно в отношении ключевых рекомендаций (см. пункт 17), хотя они не обязательно должны быть сформулированы как вопрос. Продолжая примеры, приведенные в пункте 1:

- Сколько раз в год следует измерять уровень HbA1с у больных сахарным диабетом?
- Какой должна быть ежедневная доза аспирина у больных с подтвержденным острым инфарктом миокарда?
- Снижает ли скрининг населения на предмет колоректального рака с использованием теста на скрытую кровь в кале на смертность от колоректального рака?
- Эффективен ли самоконтроль для контроля уровня глюкозы крови у пациентов с сахарным диабетом 2 типа?

ГДЕ ИСКАТЬ:

Изучите вступительные параграфы / главы, чтобы найти описание области применения и цели руководства. В некоторых случаях вопросы описаны в отдельном документе, например, в разделе поиска. Примеры разделов или глав в руководстве, где эта информация может быть найдена, включают: вопросы, цели, обоснование и актуальность.

КАК ОЦЕНИВАТЬ:

Содержимое раздела включает следующие КРИТЕРИИ:

- целевая группа населения
- вмешательства и воздействия
- сравнение (если допустимо)
- исходы
- медицинские учреждения или окружение (обстановка)

Дополнительные СООБРАЖЕНИЯ:

- Хорошо ли написан этот пункт? Являются ли описания ясными и краткими?
- Легко ли найти содержание этого пункта в руководстве?
- Достаточно ли информации по этому вопросу для инициации разработки руководства кем-либо по этой теме или понять пациентов/население и ситуацию, описанную в руководстве?

| **ОБЛАСТЬ ПРИМЕНЕНИЯ И ЦЕЛЬ**  **3. Популяция (пациенты, общество и т.д.), к которой руководство будет применяться, описана точно.** |
| --- |

| **1** Совершенно не согласен (-на) | **2** | **3** | **4** | **5** | **6** | **7** Совершенно согласен (-на) |
| --- | --- | --- | --- | --- | --- | --- |

*Комментарии:*

ОПИСАНИЕ:

Должно быть представлено ясное описание популяции (пациенты, население, и т.п.), к которой будет применяться руководство. Может быть указана возрастная категория, пол, клиническое описание и сопутствующие заболевания. Например:

- Клиническое руководство по лечению больных СД применимо только к больным инсулиннезависимым сахарным диабетом и исключает пациентов с сопутствующими сердечно – сосудистыми заболеваниями.
- Клиническое руководство по лечению депрессии применимо только к больным с диагнозом глубокой депрессии, установленным в соответствии с критериями «Руководства по диагностике и статистике психических расстройств IV издания (DSM-IV)» и не применимо к больным с психотическими симптомами и к детям.
- Руководство по скринингу рака молочной железы включает только женщин в возрасте от 50 до 70 лет, без рака в анамнезе и без рака молочной железы в семейном анамнезе.

ГДЕ ИСКАТЬ:

Изучите вступительные параграфы / главы для определения целевой группы населения в руководстве. Четко обозначенные исключения некоторых групп населения (например, детей) также должны быть описаны в этом пункте. Примеры разделов или глав в руководстве, где эта информация может быть найдена, включают: популяция пациентов, целевая популяция, соответствующие пациенты, область применения и цели.

КАК ОЦЕНИВАТЬ:

Содержимое раздела включает следующие КРИТЕРИИ:

- целевая популяция, пол и возраст
- клиническое состояние (если применимо)
- тяжесть/стадия заболевания (если применимо)
- сопутствующие заболевания (если применимо)
- исключенные популяции (если применимо)

Дополнительные СООБРАЖЕНИЯ:

- Хорошо ли написан этот пункт? Являются ли описания ясными и краткими?
- Легко ли найти содержание этого пункта в руководстве?
- Является ли информация о населении достаточно конкретизированной, чтобы соответствующие и отвечающие критериям отдельные пациенты получали бы вмешательства (действия), рекомендованные в руководстве?

| **ДОМЕН 2. УЧАСТИЕ ЗАИНТЕРЕСОВАННЫХ СТОРОН** |
| --- |

4. Группа разработчиков руководства включает специалистов из всех соответствующих профессиональных групп.

5. Были изучены взгляды и предпочтения целевой популяции (пациентов, общественности и т. д.).

6. Целевые пользователи руководства четко определены.

| **УЧАСТИЕ ЗАИНТЕРЕСОВАННЫХ СТОРОН**  **4. Группа разработчиков руководства включает специалистов из всех соответствующих профессиональных групп: .** |
| --- |

| **1** Совершенно не согласен (-на) | **2** | **3** | **4** | **5** | **6** | **7** Совершенно согласен (-на) |
| --- | --- | --- | --- | --- | --- | --- |

*Комментарии:*

ОПИСАНИЕ:

Этот пункт относится к профессионалам, которые на каких-либо этапах участвовали в разработке руководства (клинических рекомендаций). Это могут быть члены руководящей группы, команда исследователей, принимавших участие в отборе и оценке доказательств, и отдельные лица, вовлеченные в составление итоговых рекомендаций. *Этот пункт исключает лиц, осуществивших внешнее рецензирование руководства (см. пункт 13). Этот пункт исключает представление целевой аудитории (см. Пункт 5).* Должна быть представлена информация о составе, специализации и соответствующем опыте группы разработчиков руководства.

ГДЕ ИСКАТЬ:

Изучите вступительные параграфы / главы, раздел благодарностей или приложения, чтобы найти состав группы разработчиков руководства. Примеры разделов или глав в руководстве, где эта информация может быть найдена, включают: методы, список членов группы разработчиков руководства, благодарности, и приложения.

КАК ОЦЕНИВАТЬ:

Содержимое раздела включает следующие КРИТЕРИИ:

- О каждом участнике группы разработчиков руководства должна быть представлена следующая информация
- имя
- дисциплина/специализация (напр., нейрохирург, методолог)
- учреждение (например, больница Св. Петра)
- местонахождение (например, Сиэтл, США)
- описание роли участника в группе разработчиков руководства

Дополнительные СООБРАЖЕНИЯ:

- Хорошо ли написан этот пункт? Являются ли описания ясными и краткими?
- Легко ли найти содержание этого пункта в руководстве?
- Соответствуют ли участники теме и области? Потенциальные кандидаты включают соответствующих клиницистов, экспертов, исследователей, определяющих политику, клинических администраторов, и финансирующих организаций.
- Включен ли в группу разработчиков хотя бы один эксперт по методологии (например, эксперт по систематическим обзорам, эпидемиолог, статистик, библиотекарь-исследователь и т.д.)?

| **УЧАСТИЕ ЗАИНТЕРЕСОВАННЫХ СТОРОН**  **5. Были изучены взгляды и предпочтения целевой популяции (пациентов, общественности и т. д.):** |
| --- |

| **1** Совершенно не согласен (-на) | **2** | **3** | **4** | **5** | **6** | **7** Совершенно согласен (-на) |
| --- | --- | --- | --- | --- | --- | --- |

*Комментарии:*

ОПИСАНИЕ:

Информация об опыте и ожиданиях целевой популяции от здравоохранения должна информировать процесс разработки руководства (клинических рекомендаций). Существуют различные методы обеспечения того, чтобы эти точки зрения информировали разные этапы разработки руководства заинтересованными сторонами. Например, официальные консультации с пациентами / общественностью для определения приоритетных проблем, участие этих заинтересованных сторон в группе разработчиков руководства или внешнее рецензирование этими заинтересованными сторонами проекта/черновика документов. Альтернативно, информация может быть получена из интервью этих заинтересованных сторон или из литературных обзоров о ценностях пациентов, общества, их предпочтений или опыта. Должны быть представлены доказательства, что такой процесс имел место и что взгляды заинтересованных сторон были рассмотрены.

ГДЕ ИСКАТЬ:

Изучите главы о процессе разработки руководства. Примеры разделов или глав в руководстве, где эта информация может быть найдена, включают: область применения, методы, список членов группы разработчиков руководства, внешние рецензенты, и интересы целевой аудитории.

КАК ОЦЕНИВАТЬ:

Содержимое раздела включает следующие КРИТЕРИИ:

- описание стратегии сбора информации о взглядах и предпочтениях пациентов/населения (например, участие в группе разработки руководства, литературный обзор ценностей и предпочтений)
- методы, с помощью которых мнения и предпочтения были собраны (например, доказательства из литературы, опросов, фокусные группы)
- исходы/информация, собранные по пациентам/ из публичных источников
- описание того, как собранная информация была использована в процессе разработки руководства и/или составлении рекомендаций.

Дополнительные СООБРАЖЕНИЯ:

- Хорошо ли написан этот пункт? Являются ли описания ясными и краткими?
- Легко ли найти содержание этого пункта в руководстве?

| **УЧАСТИЕ ЗАИНТЕРЕСОВАННЫХ СТОРОН**  **6. Четко определена целевая аудитория (пользователи) руководства.** |
| --- |

| **1** Совершенно не согласен (-на) | **2** | **3** | **4** | **5** | **6** | **7** Совершенно согласен (-на) |
| --- | --- | --- | --- | --- | --- | --- |

*Комментарии:*

ОПИСАНИЕ:

В руководстве должна быть четко определена целевая аудитория (пользователи), чтобы читающий смог сразу определить, подходит ли ему это руководство. Например, целевой аудиторией (пользователями) руководства по боли в пояснице могут быть врачи общей практики, неврологи, хирурги-ортопеды, ревматологи и физиотерапевты.

ГДЕ ИСКАТЬ:

Изучите вступительные параграфы / главы на предмет наличия в них описания целевых пользователей руководства. Примеры разделов или глав в руководстве, где эта информация может быть найдена, включают: целевые пользователи и предполагаемый пользователь.

КАК ОЦЕНИВАТЬ:

Содержимое раздела включает следующие КРИТЕРИИ:

- четкое описание целевой аудитории (например, специалисты, семейные врачи, пациенты, клинические или учрежденческие руководители, или администраторы)
- описание того, как руководство может быть использовано целевой аудиторией (пользователями, например, для информирования клинических решений, для информирования политики здравоохранения, или для информирования стандартов помощи)

Дополнительные СООБРАЖЕНИЯ:

- Хорошо ли написан этот пункт? Являются ли описания ясными и краткими?
- Легко ли найти содержание этого пункта в руководстве?
- Соответствуют ли целевые пользователи области применения этого руководства?

| **ДОМЕН 3. ТЩАТЕЛЬНОСТЬ РАЗРАБОТКИ** |
| --- |

1. Использованы систематические методы поиска доказательств.
2. Критерии отбора доказательств четко описаны.
3. Сила и ограничения совокупности доказательств четко описаны.
4. Четко описаны методы составления и формулирования рекомендаций.
5. Польза для здоровья, побочные эффекты и риски предлагаемых подходов б были рассмотрены при составлении и формулировании рекомендаций.
6. Имеется недвусмысленная связь между рекомендациями и подкрепляющими их доказательствами.
7. Руководство было рецензировано внешними экспертами до публикации.
8. Предусмотрена процедура обновления руководства.

| **ТЩАТЕЛЬНОСТЬ РАЗРАБОТКИ**  **7. Использованы систематические методы поиска доказательств.** |
| --- |

| **1** Совершенно не согласен (-на) | **2** | **3** | **4** | **5** | **6** | **7** Совершенно согласен (-на) |
| --- | --- | --- | --- | --- | --- | --- |

*Комментарии:*

ОПИСАНИЕ:

Должна быть подробно описана стратегия поиска доказательств, включая указание терминов для поиска, источников информации и временные рамки поиска литературы. Источники информации могут включать электронные базы данных (например, MEDLINE, EMBASE, CINAHL), базы данных систематических обзоров (например, Кокрейновская библиотека, DARE), ручной поиск в журналах, рассмотрение материалов конференций, и других клинических рекомендаций (например, US National Guideline Clearinghouse, German Guidelines Clearinghouse). Стратегия поиска должна быть насколько только возможно всеобъемлющей, и выполнена без потенциальной предвзятости и достаточно детализирована, чтобы ее можно было воспроизвести.

ГДЕ ИСКАТЬ:

Изучите параграфы / главы с описанием процесса разработки руководства. В некоторых случаях стратегии поиска описывают в отдельных документах, или в приложениях к руководству. Примеры разделов или глав в руководстве, где эта информация может быть найдена, включают: методы, стратегия поиска литературы, и приложения.

КАК ОЦЕНИВАТЬ:

Содержимое раздела включает следующие КРИТЕРИИ:

- Поименованы электронные базы данных или источников доказательств, в которых был проведен поиск (например, MEDLINE, EMBASE, PsychINFO, CINAHL)
- Временные периоды проведения поиска (например, с 1 января 2004 по 31 марта 2008)
- Использованы термины для поиска (например, текстовые слова, индексируемые термины, подзаголовки)
- Включена полная стратегия поиска (например, можно найти в приложении)

Дополнительные СООБРАЖЕНИЯ:

- Хорошо ли написан этот пункт? Являются ли описания ясными и краткими?
- Легко ли найти содержание этого пункта в руководстве?
- Является ли поиск релевантным и адекватным, чтобы ответить на поставленный вопрос здоровья (здравоохранения)? (например, были использованы все релевантные базы данных и соответствующие термины поиска)
- Достаточно ли представлено информации, чтобы любому можно было бы повторить поиск?

| **ТЩАТЕЛЬНОСТЬ РАЗРАБОТКИ**  **8. Критерии отбора доказательств четко описаны.** |
| --- |

| **1** Совершенно не согласен (-на) | **2** | **3** | **4** | **5** | **6** | **7** Совершенно согласен (-на) |
| --- | --- | --- | --- | --- | --- | --- |

Комментарии:

ОПИСАНИЕ:

Должны быть указаны критерии включения/исключения доказательств, выявленных при поиске. Эти критерии должны быть детально описаны, равно как должны быть четко указаны причины, по которым доказательства включались или исключались. Например, авторы клинических рекомендаций могут принять решение включать только доказательства, полученные в рандомизированных клинических испытаниях, и исключать статьи, написанные не на английском языке.

ГДЕ ИСКАТЬ:

Изучите параграфы / главы с описанием процесса разработки руководства. В некоторых случаях критерии включения/исключения для отбора доказательств могут быть описаны в отдельных документах или в Приложении к руководству. Примеры разделов или глав в руководстве, где эта информация может быть найдена, включают: методы, литературный поиск, критерии включения/исключения, и приложения.

КАК ОЦЕНИВАТЬ:

Содержимое раздела включает следующие КРИТЕРИИ:

- описание критериев включения, в том числе:
- характеристика целевой популяции (пациента, население и проч.)
- дизайн исследования
- сравнения (если применимо)
- исходы
- язык (если применимо)
- особенности (условия) (если применимо)
- описание критерия исключения (если применимо; напр., указание *только французский язык* в критериях включения, может логически исключать утверждение *не-французский язык* в критериях исключения)

Дополнительные СООБРАЖЕНИЯ:

- Хорошо ли написан этот пункт? Являются ли описания ясными и краткими?
- Легко ли найти содержание этого пункта в руководстве?
- Есть ли обоснование для выбранных критериев включения/исключения?
- Критерии включения/исключения согласуются с вопросом здоровья (здравоохранения)?
- Есть ли основания предположить, что соответствующая литература, возможно, не была рассмотрена?

| **ТЩАТЕЛЬНОСТЬ РАЗРАБОТКИ**  **9. Сила и ограничения совокупности доказательств четко описаны.** |
| --- |

| **1** Совершенно не согласен (-на) | **2** | **3** | **4** | **5** | **6** | **7** Совершенно согласен (-на) |
| --- | --- | --- | --- | --- | --- | --- |

Комментарии:

ОПИСАНИЕ:

Должны быть представлены подтверждения, указывающие на сильные и слабые стороны доказательств. Это должно включать четкие описания – с использованием неформальных или формальных инструментов / методов – для оценки и описания риска смещения в отдельных исследованиях и/или для конкретных исходов и/или должно включать полный, не оставляющий двусмысленности комментарий по совокупности доказательств, собранных по всем исследованиям. Это может быть представлено различными способами, например: использование таблиц, комментирующих разные домены (разделы) качества; применение формального инструмента или стратегии (например, шкала Jadad, метод GRADE); или описания в тексте.

ГДЕ ИСКАТЬ:

Изучите параграфы / главы с описанием процесса разработки руководства на предмет того, как описано методологическое качество исследований (например, риск смещения). Таблицы часто используются для суммирования показателей качества. Некоторые руководства делают четкое разделение между описанием и интерпретацией доказательств, например, в разделе результатов и разделе обсуждения, соответственно.

КАК ОЦЕНИВАТЬ:

Содержимое раздела включает следующие КРИТЕРИИ:

- описание того, как доказательства были оценены на предмет смещения, и как это было интерпретировано участниками группы разработчиков руководства
- аспекты, на основе которых следует представлять описания, включают:
- дизайн исследования включен в описание доказательства
- методологические ограничения исследования (выборка, ослепление, скрытие определения в группу исследования, аналитические методы)
- рассмотрены адекватность, соответствие/обоснованность первичных и вторичных исходов
- согласованность результатов в разных исследованиях
- направление результатов в разных исследованиях
- величина пользы относительно величины наносимого вреда
- применимость на практике

Дополнительные СООБРАЖЕНИЯ:

- Хорошо ли написан этот пункт? Являются ли описания ясными и краткими?
- Легко ли найти содержание этого пункта в руководстве?
- Являются ли описания уместными, нейтральными и беспристрастными? Являются ли

описания полными?

| **ТЩАТЕЛЬНОСТЬ РАЗРАБОТКИ**  **10. Четко описаны методы составления и формулирования рекомендаций.** |
| --- |

| **1** Совершенно не согласен (-на) | **2** | **3** | **4** | **5** | **6** | **7** Совершенно согласен (-на) |
| --- | --- | --- | --- | --- | --- | --- |

Комментарии:

ОПИСАНИЕ:

Должны быть представлены методы, используемые при составлении и формулировке рекомендаций, и способов принятия окончательных решений. Например, методы могут включать систему голосования, неформальный консенсус и формальные методы консенсуса (например, методы Delphi, Glaser). Следует детально представить области, по которым имеются разногласия и методы их разрешения.

ГДЕ ИСКАТЬ:

Изучите параграфы / главы с описанием процесса разработки руководства. В некоторых случаях методы, используемые для составления и формулирования рекомендаций, описаны в отдельных документах или в приложении к руководству. Примеры разделов или глав в руководстве, где эта информация может быть найдена, включают: методы и процесс разработки руководства.

КАК ОЦЕНИВАТЬ:

Содержимое раздела включает следующие КРИТЕРИИ:

- Описание процесса разработки рекомендаций (например, шаги, используемые в модифицированной технике Delphi, процедуры голосования, которые были рассмотрены)
- Результаты процесса разработки рекомендаций (например, в какой степени консенсус был достигнут с использованием модифицированного метода Delphi, итоги голосования)
- описание того, как этот процесс повлиял на рекомендации (например, результаты метода Delphi влияют на финальные рекомендации, согласование с рекомендациями и окончательное голосование)

Дополнительные СООБРАЖЕНИЯ:

- Хорошо ли написан этот пункт? Являются ли описания ясными и четкими?
- Легко ли найти содержание этого пункта в руководстве?
- Был ли использован формальный процесс для достижения итоговых рекомендаций?
- Были ли методы соответствующими?

| **ТЩАТЕЛЬНОСТЬ РАЗРАБОТКИ**  **11. Польза для здоровья, побочные эффекты и риски предлагаемых подходов были рассмотрены при составлении и формулировании рекомендаций.** |
| --- |

| **1** Совершенно не согласен (-на) | **2** | **3** | **4** | **5** | **6** | **7** Совершенно согласен (-на) |
| --- | --- | --- | --- | --- | --- | --- |

Комментарии:

ОПИСАНИЕ:

В руководстве должны быть рассмотрены польза для здоровья, побочные эффекты и риски при составлении и формулировании рекомендаций. Например, руководство по лечению рака молочной железы может включать обсуждение общего влияния на различные конечные исходы. Они могут включать: выживание, качество жизни, неблагоприятные эффекты, и управление симптомами или обсуждения, сравнивающие один вариант лечения с другим. Должны быть доказательства того, что эти вопросы рассмотрены.

ГДЕ ИСКАТЬ:

Изучите параграфы / главы с описанием процесса разработки руководства на предмет наличия описания совокупности доказательств, их интерпретации, и трансформации в практические рекомендации. Примеры разделов или глав в руководстве, где эта информация может быть найдена, включают: методы, интерпретация, обсуждение и рекомендации.

КАК ОЦЕНИВАТЬ:

Содержимое раздела включает следующие КРИТЕРИИ:

- подтверждающие данные и отчет о пользе
- подтверждающие данные и отчет о вреде/побочных эффектах/рисках
- отчетность о балансе / компромиссе между пользой и вредом / побочными эффектами / рисками
- рекомендации отражают соображения, касающиеся как пользы, так и вреда / побочных эффектов / риска

Дополнительные СООБРАЖЕНИЯ:

- Хорошо ли написан этот пункт? Являются ли описания ясными и краткими?
- Легко ли найти содержание этого пункта в руководстве?
- Является ли обсуждение неотъемлемой частью процесса разработки рекомендаций? (т. е., происходит во время составления и формулирования рекомендации, а не после составления, как запоздалая мысль или задним числом)
- Рассмотрела ли группа разработчиков рекомендаций пользу и вред в равной степени?

| **ТЩАТЕЛЬНОСТЬ РАЗРАБОТКИ**  **12. Имеется недвусмысленная связь между рекомендациями и подкрепляющими их доказательствами.** |
| --- |

| **1** Совершенно не согласен (-на) | **2** | **3** | **4** | **5** | **6** | **7** Совершенно согласен (-на) |
| --- | --- | --- | --- | --- | --- | --- |

Комментарии:

ОПИСАНИЕ:

Должна присутствовать четко выраженная (недвусмысленная) связь между рекомендациями и доказательствами, на которых они основаны. Пользователь руководства должен иметь возможность идентифицировать компоненты приведённых доказательств, имеющих отношение к каждой рекомендации.

ГДЕ ИСКАТЬ:

Определите и изучите рекомендации руководства и текст описания совокупности доказательств, их обосновывающих. Примеры разделов или глав в руководстве, где эта информация может быть найдена, включают: рекомендации и основные доказательства.

КАК ОЦЕНИВАТЬ:

Содержимое раздела включает следующие КРИТЕРИИ:

- руководство описывает, как группа разработчиков связала и использовала доказательства для информирования рекомендаций
- каждая рекомендация связана с описанием основных доказательств / параграфом и / или списком литературы
- рекомендации связаны с резюме доказательств, с таблицами доказательств в разделе результатов руководства

Дополнительные СООБРАЖЕНИЯ:

- Существует ли соответствие между доказательствами и рекомендациями
- Легко ли найти связь между рекомендациями и подтверждающими доказательствами в руководстве?
- В случае недостаточности доказательств или составления рекомендации на основе консенсуса мнений группы разработчиков рекомендаций, а не доказательств, ясно ли это изложено и описано?

| **ТЩАТЕЛЬНОСТЬ РАЗРАБОТКИ**  **13. Руководство было рецензировано внешними экспертами до публикации.** |
| --- |

| **1** Совершенно не согласен (-на) | **2** | **3** | **4** | **5** | **6** | **7** Совершенно согласен (-на) |
| --- | --- | --- | --- | --- | --- | --- |

Комментарии:

ОПИСАНИЕ:

Руководство должно пройти внешнюю экспертизу до публикации. Рецензенты не должны быть членами группы разработчиков руководства. Рецензенты должны включать экспертов в конкретной клинической области, а также экспертов по методологии. Также могут быть включены представители целевой группы населения (пациенты, общественность). Должно быть представлено описание методологии, используемой для проведения внешней экспертизы, которое может включать список рецензентов и их принадлежность к учреждениям.

ГДЕ ИСКАТЬ:

Изучите параграфы / главы с описанием процесса разработки руководства и раздел благодарностей. Примеры разделов или глав в руководстве, где эта информация может быть найдена, включают: методы, результаты, интерпретация и благодарности.

КАК ОЦЕНИВАТЬ:

Содержимое раздела включает следующие КРИТЕРИИ:

- цель и задачи внешней экспертизы (например, улучшить качество, получить обратную связь по проекту рекомендаций, оценка применимости и осуществимости, распространение доказательств)
- методы, использованные для проведения внешней экспертизы (например, рейтинговые шкалы, открытые вопросы)
- описание внешних рецензентов (например, их число, тип рецензентов, принадлежность, трудоустройство)
- результаты / информация, собранные в ходе внешнего рецензирования (например, резюме основных результатов)
- описание того, как собранная информация использовалась для информирования процесса разработки руководства и / или формулирование рекомендаций (например, руководящая группа по разработке руководства рассмотрела результаты рецензирования при формулировании финальных рекомендаций)

Дополнительные СООБРАЖЕНИЯ:

- Хорошо ли написан этот пункт? Являются ли описания ясными и краткими?
- Легко ли найти содержание этого пункта в руководстве?
- Соответствуют ли внешние рецензенты области применения этого руководства? Было ли дано обоснование для выбора включенных рецензентов?
- Каким образом информация, полученная в ходе внешнего рецензирования, была использована группой разработчиков руководства?

| **ТЩАТЕЛЬНОСТЬ РАЗРАБОТКИ**  **14. Предусмотрена процедура обновления руководства.** |
| --- |

| **1** Совершенно не согласен (-на) | **2** | **3** | **4** | **5** | **6** | **7** Совершенно согласен (-на) |
| --- | --- | --- | --- | --- | --- | --- |

Комментарии:

ОПИСАНИЕ:

Клинические рекомендации должны отражать результаты последних исследований. Должно быть представлено четкое и ясное описание процедуры обновления рекомендаций. Например, приведено расписание обновлений, или указано, что постоянная комиссия получает результаты регулярно обновляемого литературного поиска и вносит необходимые изменения.

ГДЕ ИСКАТЬ:

Изучите вступительный параграф, другие параграфы с описанием процесса разработки руководства и заключительные параграфы. Примеры разделов или глав в руководстве, где эта информация может быть найдена, включают: методы, обновление руководства и дата выхода в свет этого руководства (клинических рекомендаций).

КАК ОЦЕНИВАТЬ:

Содержимое раздела включает следующие КРИТЕРИИ:

- Порядок обновления руководства
- Четкий временной интервал или четкие критерии для принятия решений о том, когда должно произойти обновление
- Приведена методология для процедуры обновления

Дополнительные СООБРАЖЕНИЯ:

- Хорошо ли написан этот пункт? Являются ли описания ясными и краткими?
- Легко ли найти содержание этого пункта в руководстве?

• Представлено достаточно информации, чтобы знать, когда произойдет обновление или какие критерии запускают обновление?

| **ДОМЕН 4. ЯСНОСТЬ ИЗЛОЖЕНИЯ** |
| --- |

1. Рекомендации конкретны и недвусмысленны.
2. Четко представлены различные варианты ведения соответствующего состояния или проблемы здоровья.
3. Легко выделить/определить ключевые рекомендации.

| **ЯСНОСТЬ ИЗЛОЖЕНИЯ**  **15. Рекомендации конкретны и недвусмысленны.** |
| --- |

| **1** Совершенно не согласен (-на) | **2** | **3** | **4** | **5** | **6** | **7** Совершенно согласен (-на) |
| --- | --- | --- | --- | --- | --- | --- |

Комментарии:

ОПИСАНИЕ:

Рекомендации должны содержать конкретное и точное описание того, какой вариант, в какой ситуации и в какой популяционной группе является соответствующим, согласно совокупности имеющихся доказательств.

- Пример конкретной рекомендации: детям в возрасте двух лет и старше при остром среднем отите должны быть назначены антибиотики, если боль продолжается долее трех дней, или если боль усиливается после консультации врача, несмотря на адекватное лечение обезболивающими; в этих случаях необходимо назначить амоксициллин на 7 дней (режим дозирования прилагается).
- Пример размытой (не конкретной) рекомендации: антибиотики показаны при нетипичном или осложненном течении заболевания.

Важно отметить, что в некоторых случаях доказательства не всегда однозначны и может сохраняться неопределенность в отношении лучшего варианта помощи. В этих случаях необходимо заявить об этой неопределенности в клинических рекомендациях.

ГДЕ ИСКАТЬ:

Выделите и изучите рекомендации в руководстве. Примеры разделов или глав в руководстве, где эта информация может быть найдена, включают: рекомендации и резюме.

КАК ОЦЕНИВАТЬ:

Содержимое раздела включает следующие КРИТЕРИИ:

- заявление о рекомендуемых действиях
- определение назначения или цели рекомендуемых действий (например, для улучшения качества жизни, снижения побочных эффектов)
- идентификация соответствующей популяции (например, пациенты, население)
- предупреждение или квалификационные заявления, если это применимо (например, пациенты или состояния, к которым рекомендации не будут применяться)

Дополнительные СООБРАЖЕНИЯ:

- В случае многочисленных рекомендаций (например, руководящих принципов управления), есть ли ясность о том, к кому каждая рекомендация применяется?
- Если имеется неопределенность в толковании и обсуждении доказательств, отражается ли неопределенность в рекомендации и четко ли о ней заявлено?

| **ЯСНОСТЬ ИЗЛОЖЕНИЯ**  **16. Четко представлены различные варианты ведения соответствующего состояния или проблемы здоровья.** |
| --- |

| **1** Совершенно не согласен (-на) | **2** | **3** | **4** | **5** | **6** | **7** Совершенно согласен (-на) |
| --- | --- | --- | --- | --- | --- | --- |

Комментарии:

ОПИСАНИЕ:

В руководстве, нацеленном на ведение какого-либо заболевания, должны быть представлены различные возможные варианты скрининга, профилактики, диагностики или лечения рассматриваемого состояния. Эти возможные варианты должны быть четко представлены в клинических рекомендациях.

Например, в рекомендациях по ведению депрессии могут быть предложены следующие варианты лечения:

a. лечение трициклическими антидепрессантами (TCA)

b. лечение ингибиторами обратного захвата серотонина (SSRI)

c. психотерапия

d. сочетание фармакотерапии и психотерапии.

ГДЕ ИСКАТЬ:

Изучите рекомендации и поддерживающие их доказательства. Примеры разделов или глав в руководстве, где эта информация может быть найдена, включают: резюме, рекомендации, обсуждение, варианты лечения и альтернативы лечения.

КАК ОЦЕНИВАТЬ:

Содержимое раздела включает следующие КРИТЕРИИ:

- Описание вариантов
- Описание популяции или клинической ситуации, наиболее соответствующей каждому варианту

Дополнительные СООБРАЖЕНИЯ:

- Хорошо ли написан этот пункт? Являются ли описания ясными и краткими?
- Легко ли найти содержание этого пункта в руководстве?
- Относится ли это к руководству с широкой или узкой областью применения? Этот пункт может быть более актуальным для руководств с широкой областью применения (например, посвящённых ведению состояния или проблемы, а не фокусирующихся на конкретном комплексе вмешательств по конкретному состоянию/проблеме).

| **ЯСНОСТЬ ИЗЛОЖЕНИЯ**  **17. Легко выделить/определить ключевые рекомендации.** |
| --- |

| **1** Совершенно не согласен (-на) | **2** | **3** | **4** | **5** | **6** | **7** Совершенно согласен (-на) |
| --- | --- | --- | --- | --- | --- | --- |

Комментарии:

ОПИСАНИЕ:

Пользователи должны иметь возможность легко найти самые релевантные рекомендации. Эти рекомендации отвечают на основные клинические вопросы, освещаемые клиническим руководством, и могут быть идентифицированы различными способами. Например, они могут быть кратко представлены в рамке, выделены жирным шрифтом, подчеркнуты, представлены в виде схем или алгоритмов.

ГДЕ ИСКАТЬ:

Примеры разделов или глав в руководстве, где эта информация может быть найдена, включают: резюме, заключения (выводы) и рекомендации. Некоторые руководства предоставляют отдельные резюме с ключевыми рекомендациями (например, краткое справочное руководство).

КАК ОЦЕНИВАТЬ:

Содержимое раздела включает следующие КРИТЕРИИ:

- описание рекомендаций в сводной рамке, напечатаны жирным шрифтом, подчеркнуты или представлены в виде блок-схем или алгоритмов
- конкретные рекомендации сгруппированы вместе в едином разделе

Дополнительные СООБРАЖЕНИЯ:

- Хорошо ли написан этот пункт? Являются ли описания ясными и краткими?
- Легко ли найти содержание этого пункта в руководстве?
- Выбраны ли ключевые рекомендации надлежащим образом и отражают ли они основной посыл руководства?
- Сгруппированы ли конкретные рекомендации в разделе, расположенном рядом с резюме основных доказательств?

| **ДОМЕН 5. ПРИМЕНИМОСТЬ** |
| --- |

1. В руководстве описаны способствующие факторы (фасилитаторы) и барьеры для его применения.
2. Руководство предоставляет советы и / или инструменты о том, как рекомендации могут быть реализованы на практике.
3. Рассмотрены возможные ресурсные / финансовые последствия применения рекомендаций.
4. В руководстве представлены критерии для мониторинга и / или аудита**.**

| **ПРИМЕНИМОСТЬ**  **18. В руководстве описаны способствующие факторы (фасилитаторы) и барьеры для его применения.** |
| --- |

| **1** Совершенно не согласен (-на) | **2** | **3** | **4** | **5** | **6** | **7** Совершенно согласен (-на) |
| --- | --- | --- | --- | --- | --- | --- |

Комментарии:

ОПИСАНИЕ:

На применение рекомендаций руководства могут оказать влияние существующие способствующие факторы (фасилитаторы) и барьеры.

Например:

1. Клинические рекомендации по лечению инсульта могут предписывать, чтобы координация помощи таким больным осуществлялась специализированными отделениями (инсультными блоками) и службами лечения инсульта. В конкретном регионе может быть предусмотрен специальный механизм финансирования, позволяющий создавать инсультные блоки.
2. Клинические рекомендации по лечению сахарного диабета для участковых врачей могут требовать направления таких больных для осмотра и последующего наблюдения в специализированные отделения по лечению диабета. В регионе может быть недостаточно врачей, чтобы можно было создать клиники.

ГДЕ ИСКАТЬ:

Изучите параграф / главу о распространении / внедрении руководства или, если имеется, дополнительные документы с конкретными планами или стратегиями внедрения руководства. Примеры разделов или глав в руководстве, где эта информация может быть найдена, включают: барьеры, использование руководящих принципов, и показатели качества.

КАК ОЦЕНИВАТЬ:

Содержимое раздела включает следующие КРИТЕРИИ:

- определение типов фасилитаторов и барьеров, которые были рассмотрены
- методы, с помощью которых была получена информация о фасилитаторах и барьерах на пути внедрения рекомендаций (например, отзывы или обратная связь от основных заинтересованных сторон, пилотное тестирование руководящих принципов (руководств) до широкого внедрения)
- информация/описание типов фасилитаторов и барьеров, возникших в процессе исследования (например, у практикующих специалистов есть навыки предоставлять рекомендуемую помощь, соответствующее оборудование недоступно для обеспечения всего нуждающегося населения маммографией)
- описание того, как информация повлияла на процесс разработки руководства и / или формулирование рекомендаций

Дополнительные СООБРАЖЕНИЯ:

- Хорошо ли написан этот пункт? Являются ли описания ясными и краткими?
- Легко ли найти содержание этого пункта в руководстве?
- Предоставляет ли руководство стратегию преодоления барьеров?

| **ПРИМЕНИМОСТЬ**  **19. Руководство предоставляет советы и / или инструменты о том, как рекомендации могут быть реализованы на практике.** |
| --- |

| **1** Совершенно не согласен (-на) | **2** | **3** | **4** | **5** | **6** | **7** Совершенно согласен (-на) |
| --- | --- | --- | --- | --- | --- | --- |

Комментарии:

ОПИСАНИЕ:

Чтобы руководство было эффективным, его необходимо распространять и применять с использованием дополнительных материалов. Например, они могут включать: сводный документ, краткое справочное руководство, учебные пособия, результаты пилотного теста, листовки для пациентов или компьютерную поддержку. Любые дополнительные материалы должны быть предоставлены совместно с руководством.

ГДЕ ИСКАТЬ:

Изучите параграф о распространении / внедрении руководства или, если имеется, сопутствующие материалы, которые были подготовлены для распространения и внедрения руководства. Примеры разделов или глав в руководстве, где эта информация может быть найдена, включают: инструменты, ресурсы, реализация / имплементация и приложения.

КАК ОЦЕНИВАТЬ:

Содержимое раздела включает следующие КРИТЕРИИ:

- Раздел внедрения
- Инструменты и ресурсы для облегчения внедрения / применения:
- резюме руководства
- ссылки на чек-листы (контрольные списки), алгоритмы
- ссылки на инструкции по применению
- решения, связанные с анализом барьеров (см. пункт 18)
- инструменты для повышения эффекта от способствующих факторов внедрения руководства
- результаты пилотного тестирования и извлеченные уроки
- инструкции по доступу пользователей к инструментам и ресурсам

Дополнительные СООБРАЖЕНИЯ:

- Хорошо ли написан этот пункт? Являются ли описания ясными и краткими?
- Легко ли найти содержание этого пункта в руководстве?
- Есть ли информация о разработке инструментов и процедур валидизации?

| **ПРИМЕНИМОСТЬ**  **20. Рассмотрены возможные ресурсные / финансовые последствия применения рекомендаций.** |
| --- |

| **1** Совершенно не согласен (-на) | **2** | **3** | **4** | **5** | **6** | **7** Совершенно согласен (-на) |
| --- | --- | --- | --- | --- | --- | --- |

Комментарии:

ОПИСАНИЕ:

Внедрение рекомендаций может потребовать дополнительных ресурсов. Например, может потребоваться более специализированный персонал, новое оборудование, дорогостоящая лекарственная терапия. Это может иметь финансовые последствия и сказаться на бюджете здравоохранения. Необходимо, чтобы в руководстве обсуждалось возможное влияние внедрения рекомендаций на ресурсы.

ГДЕ ИСКАТЬ:

Изучите параграф о распространении / внедрении руководства или, если имеется, дополнительные документы с конкретным планом или стратегией внедрения / имплементации руководства. Некоторые руководства представляют информацию о стоимости и влиянии внедрения на затраты в пунктах, в которых обсуждаются доказательства или решения, лежащие в основе рекомендаций. Примеры разделов или глав в руководстве, где эта информация может быть найдена, включают: методы, полезность затрат, эффективность затрат, затраты на приобретение и последствия для бюджетов.

КАК ОЦЕНИВАТЬ:

Содержимое раздела включает следующие КРИТЕРИИ:

- определение видов информации о стоимости / затратах, которые были рассмотрены (например, экономические оценки, стоимость приобретения лекарств)
- методы, с помощью которых запрашивается информация о стоимости / затратах (например, экономист в области здравоохранения был в группе разработчиков, использование оценки технологий здравоохранения для конкретных препаратов и т. д.)
- информация / описание информации о стоимости / затратах, полученной в результате исследования (удельные затраты на приобретение лекарств на курс лечения)
- описание того, как собранная информация использовалась для информирования процесса разработки руководства и / или составления, формулирования рекомендаций

Дополнительные СООБРАЖЕНИЯ:

- Хорошо ли написан этот пункт? Являются ли описания ясными и краткими?
- Легко ли найти содержание этого пункта в руководстве?
- Были привлечены соответствующие эксперты для нахождения и анализа информации о стоимости / затратах?

| **ПРИМЕНИМОСТЬ**  **21. В руководстве представлены критерии для мониторинга и / или аудита.** |
| --- |

| **1** Совершенно не согласен (-на) | **2** | **3** | **4** | **5** | **6** | **7** Совершенно согласен (-на) |
| --- | --- | --- | --- | --- | --- | --- |

Комментарии:

ОПИСАНИЕ:

Оценка применения рекомендаций руководства может способствовать их продолжающемуся использованию, или повысить эффективность их использования. Это требует четко определенных критериев, полученных из ключевых рекомендаций руководства. Критерии могут включать в себя измерители процесса, поведенческие показатели, клинические или медицинские показатели исхода.

Примеры критериев мониторинга и аудита:

- HbA1c должен быть <8.0 %.
- Уровень диастолического давления должен быть <95 мм рт. ст.
- 80% населения старше 50 лет должно подвергаться скринингу на предмет колоректального рака с использованием теста на скрытую кровь в кале.
- Если жалобы при остром среднем отите продолжаются более трех дней, должен быть назначен амоксициллин.

ГДЕ ИСКАТЬ:

Изучите параграф / главу, посвященную аудиту или мониторингу использования руководства или, если таковые имеются, дополнительные документы с конкретными планами или стратегиями по оценке руководства. Примеры разделов или глав в руководстве, где эта информация может быть найдена, включают: рекомендации, индикаторы качества и критерии для аудита.

КАК ОЦЕНИВАТЬ:

Содержимое раздела включает следующие КРИТЕРИИ:

- определение критериев для оценки внедрения руководства или приверженности к рекомендациям
- критерии для оценки воздействия / эффектов внедрения руководства
- советы по частоте и интервалам измерения
- описание или рабочее определение того, как должны быть измерены критерии

Дополнительные СООБРАЖЕНИЯ:

- Хорошо ли написан этот пункт? Являются ли описания ясными и краткими?
- Легко ли найти содержание этого пункта в руководстве?
- Предоставлены ли различные критерии, включая измерители процесса, поведенческие меры и клинические или медицинские исходы?

| **ДОМЕН 6. РЕДАКЦИОННАЯ НЕЗАВИСИМОСТЬ** |
| --- |

1. Взгляды финансирующей организации не повлияли на содержание руководства
2. Конкурирующие интересы членов группы по разработке руководства были документированы и учтены:

| **РЕДАКЦИОННАЯ НЕЗАВИСИМОСТЬ**  **22. Взгляды финансирующей организации не повлияли на содержание руководства.** |
| --- |

| **1** Совершенно не согласен (-на) | **2** | **3** | **4** | **5** | **6** | **7** Совершенно согласен (-на) |
| --- | --- | --- | --- | --- | --- | --- |

Комментарии:

ОПИСАНИЕ:

Многие клинические рекомендации разрабатываются за счет финансирования из внешних источников (например, правительство, профессиональные ассоциации, благотворительные фонды, фармацевтические компании). Финансирование может покрывать все расходы, связанные с разработкой, или их часть (например, печать клинических рекомендаций). Должно присутствовать полное детальное заявление о том, что взгляды или интересы финансирующей организации не повлияли на окончательную версию рекомендаций.

ГДЕ ИСКАТЬ

Изучите параграфы / главы с описанием процесса разработки руководства или раздел благодарностей. Примеры разделов или глав в руководстве, где эта информация может быть найдена, включают: отказ от ответственности (дисклеймер) и источники финансирования.

КАК ОЦЕНИВАТЬ

Содержимое раздела включает следующие КРИТЕРИИ:

- Наименование финансирующей организации или источника финансирования (или полное и детальное заявление об отсутствии финансирования)
- Заявление о том, что финансирующая организация не повлияла на содержание руководства

Дополнительные СООБРАЖЕНИЯ:

- Хорошо ли написан этот пункт? Являются ли описания ясными и краткими?
- Легко ли найти содержание этого пункта в руководстве?
- Как группа по разработке руководства учитывала потенциальное влияние со стороны финансирующей организации?

| **РЕДАКЦИОННАЯ НЕЗАВИСИМОСТЬ**  **23. Конкурирующие интересы членов группы по разработке руководства были документированы и учтены.** |
| --- |

| **1** Совершенно не согласен (-на) | **2** | **3** | **4** | **5** | **6** | **7** Совершенно согласен (-на) |
| --- | --- | --- | --- | --- | --- | --- |

Комментарии:

ОПИСАНИЕ:

Существуют обстоятельства, при которых у членов группы по разработке руководства могут быть конкурирующие интересы. Это применимо, например, к члену группы разработчиков, научные исследования которого, совпадающие с тематикой руководства, также финансируются фармацевтической компанией. Должно присутствовать в явном виде полное и детальное заявление о том, что все члены группы заявили о том, имелись ли у них какие-либо конкурирующие интересы.

ГДЕ ИСКАТЬ

Изучите параграфы / главы с описанием группы по разработке руководства или раздел благодарностей. Примеры разделов или глав в руководстве, где эта информация может быть найдена, включают: методы, конфликт интересов, состав группы разработчиков руководства, приложение.

КАК ОЦЕНИВАТЬ

Содержимое раздела включает следующие КРИТЕРИИ:

- Рассмотрено описание типов конкурирующих интересов
- Методы, с помощью которых потенциальные конкурирующие интересы были выявлены (описаны)
- описание конкурирующих интересов
- описание того, как конкурирующие интересы повлияли на процесс разработки руководства и формулирование рекомендаций

Дополнительные СООБРАЖЕНИЯ:

- Хорошо ли написан этот пункт? Являются ли описания ясными и краткими?
- Легко ли найти содержание этого пункта в руководстве?
- Какие меры были приняты для минимизации влияния конкурирующих интересов на разработку руководства и формулирование рекомендаций?

| **ОБЩАЯ ОЦЕНКА РУКОВОДСТВА** |
| --- |

**ОБЩАЯ ОЦЕНКА РУКОВОДСТВА**

**На каждый вопрос, пожалуйста, выберите ответ, который лучше всего характеризует оцененное руководство:**

| **1. Оцените общее качество этого руководства.** |
| --- |

| **1** Совершенно не согласен (-на) | **2** | **3** | **4** | **5** | **6** | **7** Совершенно согласен (-на) |
| --- | --- | --- | --- | --- | --- | --- |

| **2. Я бы рекомендовал это руководство для использования.** |
| --- |

| **Да** |  |
| --- | --- |
| **Да, с поправками** |  |
| **Нет** |  |

**ЗАМЕТКИ**

|  |
| --- |

**ОПИСАНИЕ:**

Общая оценка требует от пользователя AGREE II вынесения суждения относительно качества руководства, с учетом пунктов оценки, рассмотренных в процессе анализа.

| **ИНСТРУМЕНТ AGREE II** |
| --- |

| **ДОМЕН 1. ОБЛАСТЬ ПРИМЕНЕНИЯ И ЦЕЛЬ** |
| --- |

| 1. Общая (-ие) цель (-и) клинического руководства подробно описана (-ы).  \| **1** Совершенно не согласен (-на) \| **2** \| **3** \| **4** \| **5** \| **6** \| **7** Совершенно согласен (-на) \| \| --- \| --- \| --- \| --- \| --- \| --- \| --- \|   Комментарии:   1. Вопросы здоровья / здравоохранения, рассматриваемые в руководстве, подробно описаны.  \| **1** Совершенно не согласен (-на) \| **2** \| **3** \| **4** \| **5** \| **6** \| **7** Совершенно согласен (-на) \| \| --- \| --- \| --- \| --- \| --- \| --- \| --- \|   Комментарии:   1. Популяция (пациенты, общество и т.д.), к которой руководство будет применяться, описана точно.  \| **1** Совершенно не согласен (-на) \| **2** \| **3** \| **4** \| **5** \| **6** \| **7** Совершенно согласен (-на) \| \| --- \| --- \| --- \| --- \| --- \| --- \| --- \|   Комментарии: |
| --- | --- | --- | --- | --- | --- | --- | --- | --- | --- | --- | --- | --- | --- | --- | --- | --- | --- | --- | --- | --- | --- |

| **ДОМЕН 2. УЧАСТИЕ ЗАИНТЕРЕСОВАННЫХ СТОРОН** |
| --- |

| 1. Группа разработчиков руководства включает специалистов из всех соответствующих профессиональных групп.  \| **1** Совершенно не согласен (-на) \| **2** \| **3** \| **4** \| **5** \| **6** \| **7** Совершенно согласен (-на) \| \| --- \| --- \| --- \| --- \| --- \| --- \| --- \|   Комментарии:   1. Были изучены взгляды и предпочтения целевой популяции (пациентов, общественности и т. д.).  \| **1** Совершенно не согласен (-на) \| **2** \| **3** \| **4** \| **5** \| **6** \| **7** Совершенно согласен (-на) \| \| --- \| --- \| --- \| --- \| --- \| --- \| --- \|   Комментарии:  6. Целевые пользователи руководства четко определены.   \| **1** Совершенно не согласен (-на) \| **2** \| **3** \| **4** \| **5** \| **6** \| **7** Совершенно согласен (-на) \| \| --- \| --- \| --- \| --- \| --- \| --- \| --- \|   Комментарии: |
| --- | --- | --- | --- | --- | --- | --- | --- | --- | --- | --- | --- | --- | --- | --- | --- | --- | --- | --- | --- | --- | --- |

| **ДОМЕН 3. ТЩАТЕЛЬНОСТЬ РАЗРАБОТКИ** |
| --- |

| 7. Использованы систематические методы поиска доказательств.   \| **1** Совершенно не согласен (-на) \| **2** \| **3** \| **4** \| **5** \| **6** \| **7** Совершенно согласен (-на) \| \| --- \| --- \| --- \| --- \| --- \| --- \| --- \|   Комментарии:  8. Критерии отбора доказательств четко описаны.   \| **1** Совершенно не согласен (-на) \| **2** \| **3** \| **4** \| **5** \| **6** \| **7** Совершенно согласен (-на) \| \| --- \| --- \| --- \| --- \| --- \| --- \| --- \|   Комментарии:  9. Сила и ограничения совокупности доказательств четко описаны.   \| **1** Совершенно не согласен (-на) \| **2** \| **3** \| **4** \| **5** \| **6** \| **7** Совершенно согласен (-на) \| \| --- \| --- \| --- \| --- \| --- \| --- \| --- \|   Комментарии: |
| --- | --- | --- | --- | --- | --- | --- | --- | --- | --- | --- | --- | --- | --- | --- | --- | --- | --- | --- | --- | --- | --- |

| **ДОМЕН 3. ТЩАТЕЛЬНОСТЬ РАЗРАБОТКИ продолжение** |
| --- |

| 1. Четко описаны методы составления и формулирования рекомендаций.  \| **1** Совершенно не согласен (-на) \| **2** \| **3** \| **4** \| **5** \| **6** \| **7** Совершенно согласен (-на) \| \| --- \| --- \| --- \| --- \| --- \| --- \| --- \|   Комментарии:   1. Польза для здоровья, побочные эффекты и риски предлагаемых подходов были рассмотрены при составлении и формулировании рекомендаций.  \| **1** Совершенно не согласен (-на) \| **2** \| **3** \| **4** \| **5** \| **6** \| **7** Совершенно согласен (-на) \| \| --- \| --- \| --- \| --- \| --- \| --- \| --- \|   Комментарии:   1. Имеется недвусмысленная связь между рекомендациями и подкрепляющими их доказательствами.  \| **1** Совершенно не согласен (-на) \| **2** \| **3** \| **4** \| **5** \| **6** \| **7** Совершенно согласен (-на) \| \| --- \| --- \| --- \| --- \| --- \| --- \| --- \|   Комментарии: |
| --- | --- | --- | --- | --- | --- | --- | --- | --- | --- | --- | --- | --- | --- | --- | --- | --- | --- | --- | --- | --- | --- |

| **ДОМЕН 3. ТЩАТЕЛЬНОСТЬ РАЗРАБОТКИ продолжение** |
| --- |

| 1. Руководство было рецензировано внешними экспертами до публикации.  \| **1** Совершенно не согласен (-на) \| **2** \| **3** \| **4** \| **5** \| **6** \| **7** Совершенно согласен (-на) \| \| --- \| --- \| --- \| --- \| --- \| --- \| --- \|  1. Предусмотрена процедура обновления руководства.  \| **1** Совершенно не согласен (-на) \| **2** \| **3** \| **4** \| **5** \| **6** \| **7** Совершенно согласен (-на) \| \| --- \| --- \| --- \| --- \| --- \| --- \| --- \|   Комментарии:  Комментарии: |
| --- | --- | --- | --- | --- | --- | --- | --- | --- | --- | --- | --- | --- | --- | --- |

| **ДОМЕН 4. ЯСНОСТЬ ИЗЛОЖЕНИЯ** |
| --- |

| 1. Рекомендации конкретны и недвусмысленны.  \| **1** Совершенно не согласен (-на) \| **2** \| **3** \| **4** \| **5** \| **6** \| **7** Совершенно согласен (-на) \| \| --- \| --- \| --- \| --- \| --- \| --- \| --- \|   Комментарии:   1. Четко представлены различные варианты ведения соответствующего состояния или проблемы здоровья.  \| **1** Совершенно не согласен (-на) \| **2** \| **3** \| **4** \| **5** \| **6** \| **7** Совершенно согласен (-на) \| \| --- \| --- \| --- \| --- \| --- \| --- \| --- \|   Комментарии:   1. Легко выделить/определить ключевые рекомендации.  \| **1** Совершенно не согласен (-на) \| **2** \| **3** \| **4** \| **5** \| **6** \| **7** Совершенно согласен (-на) \| \| --- \| --- \| --- \| --- \| --- \| --- \| --- \|   Комментарии: |
| --- | --- | --- | --- | --- | --- | --- | --- | --- | --- | --- | --- | --- | --- | --- | --- | --- | --- | --- | --- | --- | --- |

| **ДОМЕН 5. ПРИМЕНИМОСТЬ** |
| --- |

| 1. В руководстве описаны способствующие факторы (фасилитаторы) и барьеры для его применения.  \| **1** Совершенно не согласен (-на) \| **2** \| **3** \| **4** \| **5** \| **6** \| **7** Совершенно согласен (-на) \| \| --- \| --- \| --- \| --- \| --- \| --- \| --- \|   Комментарии:   1. Руководство предоставляет советы и / или инструменты о том, как рекомендации могут быть реализованы на практике.  \| **1** Совершенно не согласен (-на) \| **2** \| **3** \| **4** \| **5** \| **6** \| **7** Совершенно согласен (-на) \| \| --- \| --- \| --- \| --- \| --- \| --- \| --- \|   Комментарии:   1. Рассмотрены возможные ресурсные / финансовые последствия применения рекомендаций.   Комментарии: |
| --- | --- | --- | --- | --- | --- | --- | --- | --- | --- | --- | --- | --- | --- | --- |

| **ДОМЕН 5. ПРИМЕНИМОСТЬ продолжение** |
| --- |

| 1. В руководстве представлены критерии для мониторинга и / или аудита**.**  \| **1** Совершенно не согласен (-на) \| **2** \| **3** \| **4** \| **5** \| **6** \| **7** Совершенно согласен (-на) \| \| --- \| --- \| --- \| --- \| --- \| --- \| --- \|   Комментарии: |
| --- | --- | --- | --- | --- | --- | --- | --- |

| **ДОМЕН 6. РЕДАКЦИОННАЯ НЕЗАВИСИМОСТЬ** |
| --- |

| 1. Взгляды финансирующей организации не повлияли на содержание руководства  \| **1** Совершенно не согласен (-на) \| **2** \| **3** \| **4** \| **5** \| **6** \| **7** Совершенно согласен (-на) \| \| --- \| --- \| --- \| --- \| --- \| --- \| --- \|   Комментарии:   1. Конкурирующие интересы членов группы по разработке руководства были документированы и учтены:  \| **1** Совершенно не согласен (-на) \| **2** \| **3** \| **4** \| **5** \| **6** \| **7** Совершенно согласен (-на) \| \| --- \| --- \| --- \| --- \| --- \| --- \| --- \|   Комментарии: |
| --- | --- | --- | --- | --- | --- | --- | --- | --- | --- | --- | --- | --- | --- | --- |

**ОБЩАЯ ОЦЕНКА РУКОВОДСТВА**

**На каждый вопрос, пожалуйста, выберите ответ, который лучше всего характеризует оцененное руководство:**

| **1. Оцените общее качество этого руководства.** |
| --- |

| **1** Самое низкое возможное качество | **2** | **3** | **4** | **5** | **6** | **7** Наивысшее возможное качество |
| --- | --- | --- | --- | --- | --- | --- |

| **2. Я бы рекомендовал это руководство для использования.** |
| --- |

| **Да** |  |
| --- | --- |
| **Да, с поправками** |  |
| **Нет** |  |

**ПРИМЕЧАНИЯ**

|  |
| --- |
