# Supplementary material for: Assessing methodological quality of Russian clinical practice guidelines and introducing AGREE II instrument in Russia
Source: PLoS One. 2018 Sep 11;13(9):e0203328. doi: 10.1371/journal.pone.0203328 (PMC6133363; doi:10.1371/journal.pone.0203328)
Supplement: S1 Fig — (PDF) [file pone.0203328.s005.pdf]

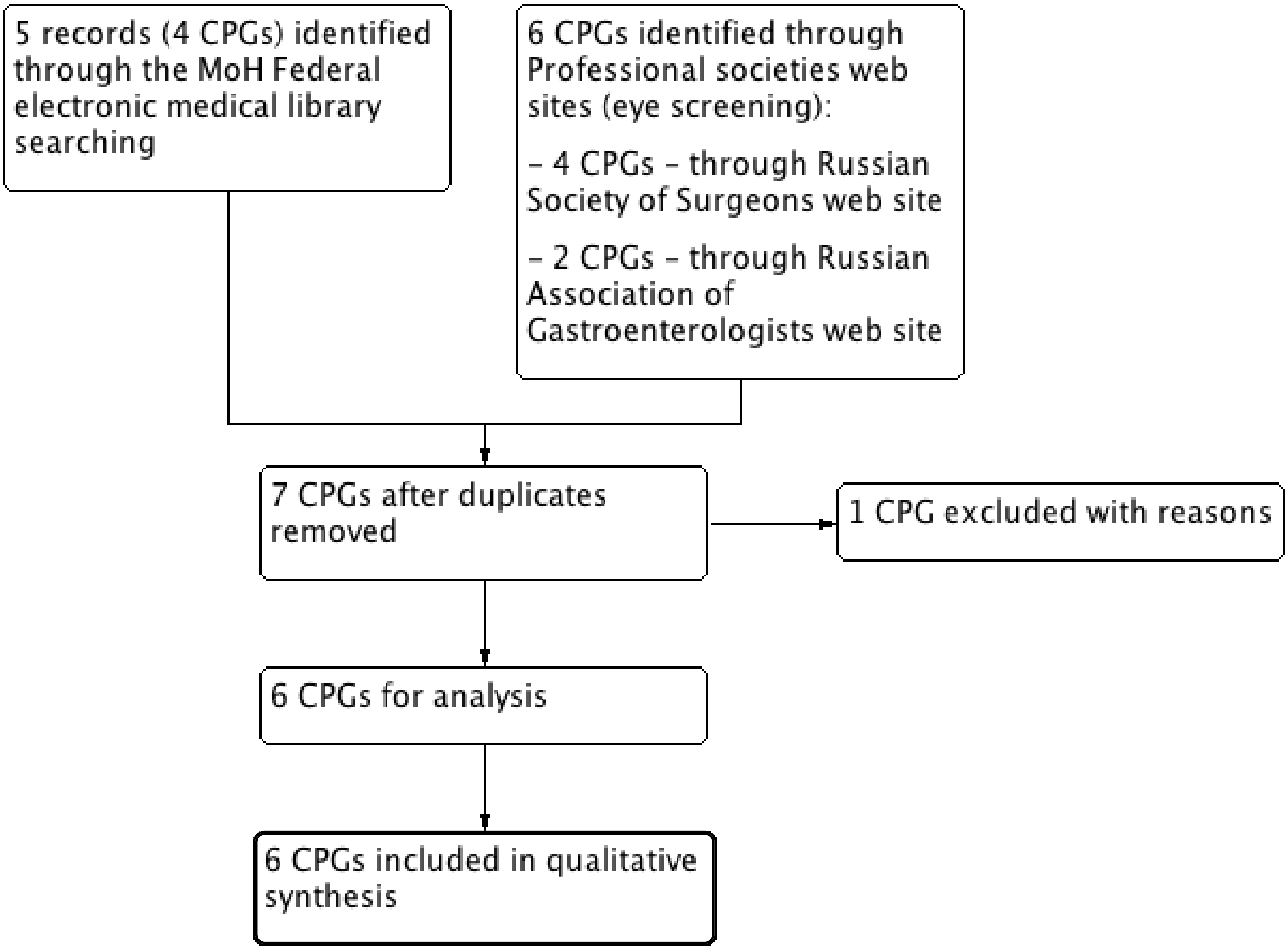
5 records (4 CPGs) identified through the MoH Federal electronic medical library searching
6 CPGs identified through Professional societies web sites (eye screening):
– 4 CPGs – through Russian Society of Surgeons web site
– 2 CPGs – through Russian Association of Gastroenterologists web site
7 CPGs after duplicates removed
1 CPG excluded with reasons
6 CPGs for analysis
6 CPGs included in qualitative synthesis
