# Supplementary figures and images for: Assessing methodological quality of Russian clinical practice guidelines and introducing AGREE II instrument in Russia
Source: PLoS One. 2018 Sep 11;13(9):e0203328. doi: 10.1371/journal.pone.0203328 (PMC6133363; doi:10.1371/journal.pone.0203328)

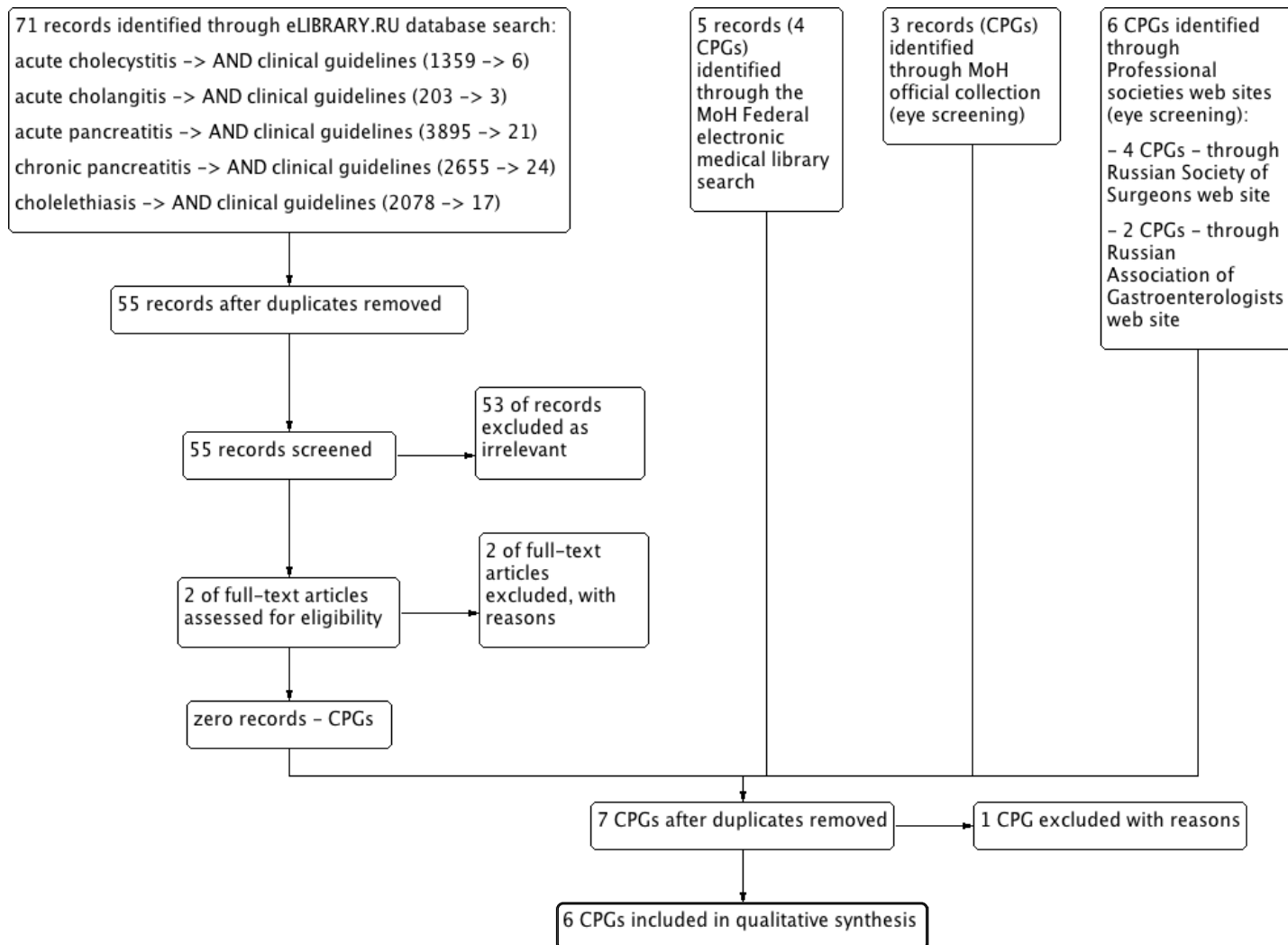

Supplement: S2 Fig — (PDF) [file pone.0203328.s006.pdf]
