# Supplementary material for: Assessing methodological quality of Russian clinical practice guidelines and introducing AGREE II instrument in Russia
Source: PLoS One. 2018 Sep 11;13(9):e0203328. doi: 10.1371/journal.pone.0203328 (PMC6133363; doi:10.1371/journal.pone.0203328)
Supplement: S1 Appendix — (DOCX) [file pone.0203328.s007.docx]

**S1 Appendix. Description of the level of expertise of the Expert Panel**

The Expert Panel comprised of gentlemen representing the age groups from forty to over sixty, with high level of specialised expertise in abdominal surgery.

- Professor L.E. Slavin, MD, PhD, DS, specializes in endoscopic and endocrine surgery. He is the Head of Surgery clinic of the “State Autonomous Healthcare Institution “Inter-regional Clinical Diagnostic Center””. He is an active member of the European Association of Endoscopic Surgeons. His PhD dissertation was about cholelithiasis in obese patients. He is the Surgeon of the highest category, he is a Professor at the Department of Surgery of the Kazan State Medical Academy.
- Professor L.V. Kim, MD, PhD, DS, specializes in hepatopancreatobiliary pathology. He is the Deputy Chief Medical Officer of Kaliningrad Emergency Hospital. He is an active member of the International Association of Hepatopancreatic Surgeons of CIS Countries. His PhD dissertation was about hepatic-pancreatic-biliary pathology. He is a Professor, lecturing at the Department of Surgical Disciplines, Baltic Federal University. I. Kant, Medical Institute;
- Dr. G.L. Kuznetsov, MD, PhD, specializes in hepatopancreatobiliary pathology. He is the Head of the surgical department of the Regional Clinical Hospital of the Kaliningrad Region, an active member of the International Association of Hepatopancreatic Surgeons of CIS Countries. His PhD dissertation was on pancreatic necrosis. He is a lecturer at the Department of Surgical Disciplines, Baltic Federal University. I. Kant, medical institute.
- Associate Professor I.Z. Weissbein, MD, PhD, specializes in general surgery. He is the Deputy Chief Medical Officer of the Regional Clinical Hospital of the Kaliningrad Region; the Head of the Kaliningrad Society of Surgeons. His PhD dissertation was about bleeding ulcers in the gastrointestinal tract. He is the Surgeon of the highest category. He is the associate professor of the Department of Surgical Disciplines, Baltic Federal University. I. Kant, Medical Institute;
