# Supplementary material for: Assessing methodological quality of Russian clinical practice guidelines and introducing AGREE II instrument in Russia
Source: PLoS One. 2018 Sep 11;13(9):e0203328. doi: 10.1371/journal.pone.0203328 (PMC6133363; doi:10.1371/journal.pone.0203328)
Supplement: S1 Table — (DOCX) [file pone.0203328.s008.docx]

**S1 Table. Reasons for exclusion**

|  | Reference | Reason |
| --- | --- | --- |
| Journal paper 1 from eLIBRARY search 2018 | Кучерявый Ю.А., Андреев Д.Н. Краткие положения национальных рекомендаций российской гастроэнтерологической ассоциации по диагностике и лечению хронического панкреатита. Альманах клинической медицины. 2014. № 33. С. 15-22. | Abbreviated version of the Russian Association of gastroenterologists guidelines for chronic pancreatitis, not possible to apply AGREE instrument, therapeutic, not surgical approaches |
| Journal paper 2 from eLIBRARY search 2018 | Ильченко А.А. Рекомендации научного общества гастроэнтерологов России по диагностике и лечению желчнокаменной болезни* и краткие комментарии. Consilium Medicum. 2012. Т. 14. № 8. С. 21-29. | Outdated, before 2013 |
| Journal paper on the website of the RAG | Клиническая рекомендация:"Хронический панкреатит у взрослых" 2013 (updated in 2016) | Guidelines of the Russian Association of gastroenterologists (RAG) guidelines for chronic pancreatitis: therapeutic, not surgical approaches |
